# Analytical solution of a microrobot-blood vessel interaction model

**DOI:** 10.1007/s11071-024-10318-2

**Published:** 2024-10-14

**Authors:** Gengxiang Wang, Andrew Bickerdike, Yang Liu, Antoine Ferreira

**Affiliations:** 1 Exeter Small-Scale Robotics Laboratory, Engineering Department, University of Exeter, Exeter, EX4 4QF UK; 2 School of Mechanical and Electrical Engineering, Xi’an University of Architecture and Technology, Xi’an, 710055 Shaanxi China; 3 Laboratoire PRISME, INSA Centre Val de Loire, 18000 Bourges, France

**Keywords:** Impact oscillator, Vibro-impact dynamics, Microrobot, Analytical solution, Cancer detection

## Abstract

This study develops a dynamics model of a microrobot vibrating in a blood vessel aiming to detect potential cancer metastasis. We derive an analytical solution for microrobot’s motion, considering interactions with the vessel walls modelled by a linear spring-dashpot and a constant damping value for blood viscosity. The model facilitates instantaneous state transitions of the microrobot, such as contact with the vessel wall and free motion within the fluid. Amplitudes and phase angles from the transient solutions of dynamics model of the microrobot are solved at arbitrary moments, providing insights into its transient dynamics. The analytical solution of the proposed system is validated by experimental data, serving as a benchmark to examine the influence of pertinent parameters on microrobot’s dynamic response. It is found that the contact force transmitted to the vessel wall, assessed by system’s transmissibility function dependent on damping and frequency ratios, decreases with increasing damping ratio and intensifies when the frequency ratio is below $\sqrt 2$. At the frequency ratio is equal to 1, resonance phenomenon is dominated by the magnification factor linked to the damping ratio, increasing the amplitude of resonance as damping decreases. Finally, different sets of system parameters, including excitation frequency and magnitude, fluid damping, vessel wall’s stiffness and damping, reveal multi-periodic motions and fake collision of the microrobot with the vessel wall. Simulation results imply that these phenomena are minimally affected by vessel wall’s stiffness but are significantly influenced by other parameters, such as fluid damping coefficient and damping coefficient of the blood vessel wall. This research provides a robust theoretical foundation for developing control strategies for microrobots aimed at detecting cancer metastasis.

## Introduction

**Fig. 1:**
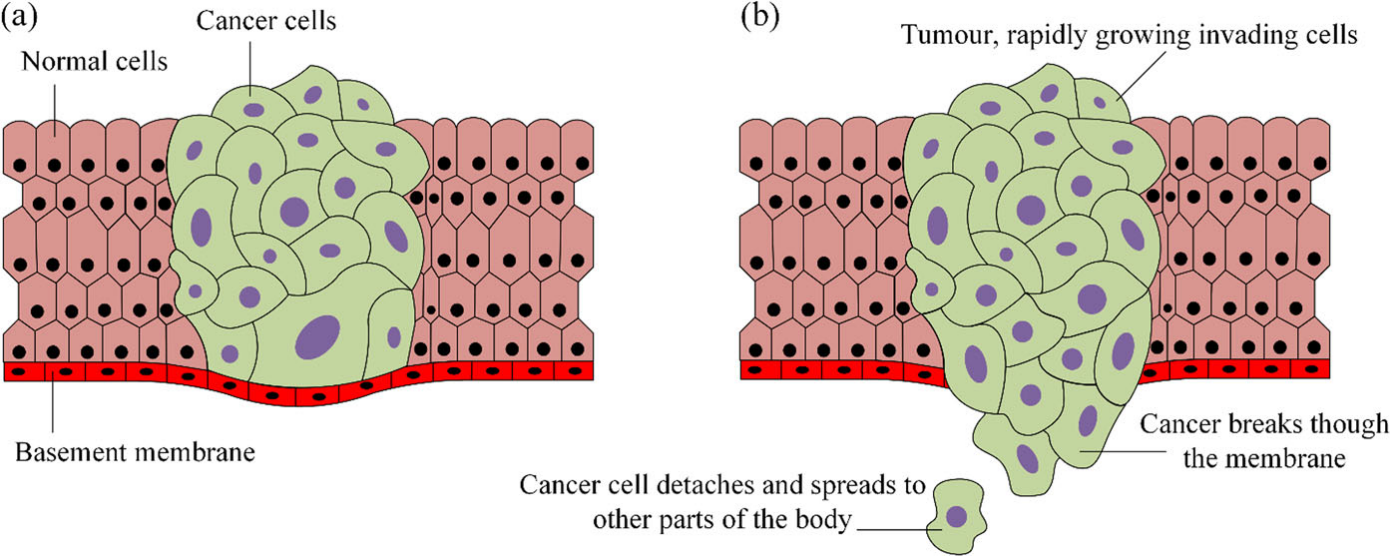
**a** As tumors grow and cancer cells accumulate increasing genetic changes, they penetrate the tissue wall and become metastatic. **b** Some cells gain the ability to detach from the primary tumor, enter the bloodstream or lymphatic system, migrate to distant locations in the body, and form new tumors. These secondary sites are typically distant lymph nodes or organs such as the liver and lungs [1]

Cancer metastasis refers to the complex process by which cancer cells spread from the original (primary) tumor to distant organs or tissues in the body, forming secondary (metastatic) tumors [1]. This spread can occur via the bloodstream, lymphatic system, or by directly invading neighboring tissues. Initially, cancer cells at the primary site grow and invade surrounding tissues, breaking through the extracellular matrix, see Fig. 1. Once they invade local tissues, cancer cells enter the bloodstream or lymphatic system [2]. As they circulate throughout the body, these cells eventually exit the bloodstream or lymphatic system, penetrating blood vessel walls to reach and establish themselves in new tissues, forming secondary tumors. Therefore, there is an urgent need to develop new techniques for detecting early metastasis or assessing the metastatic potential of tumors at the time of diagnosis, before making treatment decisions. However, detecting early cancer metastasis is highly challenging due to the complex mechanical forces in the tumor microenvironment, such as the interaction of solid and fluid stresses. To detect or prevent potential cancer cell metastasis, microrobots [3] were designed to navigate within the human body, detecting cancer cells traveling through the bloodstream or lymphatic system. These microrobots travel through blood vessels to gather information, detect cancer cells [4], or deliver diagnostic materials [5]. As the mechanical characteristics of the vessel wall change at different stages of invasion, we will use a vibrating microrobot within the blood vessel to study its dynamic responses. Based on the analysis of these dynamic responses, we can facilitate early detection of potential metastasis before cancer cells spread to distant sites.

Magnetically actuated nanoparticle microrobots [2, 6, 7] have shown significant potential across diverse fields over the past decades, including biomedical applications [8], environmental engineering [9], chemical engineering [10], geosciences [11], biomanufacturing [12, 13], drug delivery [14, 15], and materials science [16]. These microrobots represent a versatile and promising technology, driving innovation and opening new avenues for research and development across multiple disciplines. Recently, this topic has garnered considerable scholarly attention, leading to a substantial body of systematic research focused on exploring their unique properties and potential applications. For example, Li et al. [17] identified three major challenges hindering the practical use of swimming microrobots for therapeutic agent delivery: biocompatibility and biodegradability concerns, efficient locomotion in major blood vessels, and prolonged retention of the microrobots. Bozuyuk et al. [18] examined the upstream locomotion potential of surface microrollers in blood vessels using computational fluid dynamics (CFD) analyses, simplifying CFD conditions to reduce computational costs, especially in large blood vessels. This study assessed the viability and performance of surface microrollers in navigating against blood flow, offering insights into their potential biomedical applications. Sungwoong et al. [7] developed a soft microrobot to enhance the steerability of intravascular guidewires. They created a mathematical model to analyze angular deformation and derived a feed-forward method to map and guide the microrobot’s deformation. This soft microrobot, attached to the tip of a guidewire, is magnetically controlled by adjusting the direction and intensity of an external magnetic field, potentially improving the precision and ease of navigating medical instruments through vascular systems during minimally invasive procedures. Jeong et al. [19] introduced an intravascular therapeutic microrobot system that uses an electromagnetic actuation system with bi-plane X-ray devices, enabling remote control within blood vessels. The system can be utilized in various fields, including intravascular robotics, brain surgery, active capsule endoscopy, active drug delivery, and minimally invasive treatments, significantly enhancing precision and control in medical procedures. Sun et al. [20] proposed an adaptive replanning and control strategy for magnetic multi-microrobot systems aimed at minimally invasive intraarterial therapy or biosensing. They developed an extended adaptive approach to ensure asymptotic stability, allowing real-time replanning, and enhancing the flexibility and robustness of these systems in clinical applications. Talaśka and Ferreira [21] proposed a method to identify the nature and magnitude of forces required to mobilize micro- and nanoparticles on blood vessel walls using CFD and finite element method. This approach provides a deeper understanding of particle interactions with blood vessel walls, leading to more effective control over particle movement and offering a valuable tool for planning and designing drug delivery strategies via micro- and nanoparticles. However, the existing literature contains very limited studies on particle interactions with blood vessel walls. To address this gap, the present study will establish a microrobot-blood vessel interaction model which is simple enough to facilitate extensive dynamic analyses.

Our proposed model for microrobot-blood vessel interaction is based on traditional impact oscillators with elastic constraints, as seen in studies such as [22, 23]. In this model, the vibrating mass and the elastic constraints represent the microrobot and the vessel walls, respectively. Extensive research exists on impact oscillators. For example, James et al. [24] introduced a novel experimental technique and developed a mathematical model of a damped impact oscillator with a one-sided elastic constraint, aiming to analyze the stability of its periodic orbits. This enhanced the understanding of impact oscillator behavior under different conditions, potentially leading to more robust designs and applications involving impact dynamics. Zhang et al. [25] investigated the use of a delayed feedback controller with a constant delay to manage coexisting attractors in a periodically forced impact oscillator with a bidirectional drift. Their goal was to improve its progression efficiency by transitioning the system to the attractor with the highest progression speed. Yin et al. [26] explored the dynamics of a vibro-impact self-propelled capsule robot, an impact oscillator with a two-sided elastic constraint exhibiting a bidirectional drift, interacting with two types of folds, the circular fold and the cone fold, within the small intestine for endoscopic diagnosis. This study provided valuable insights into controlling the robot’s locomotion for detecting bowel cancer through different circular folds and small bowel tumors. Guo et al. [27] investigated discontinuity-induced grazing and adding-sliding bifurcations in a piecewise-smooth impact oscillator subjected to bidirectional drifts, utilizing both numerical and analytical methods. They verified their analytical approach numerically with path-following techniques designed for piecewise-smooth dynamical systems. Using the continuation platform COCO, they identified the onset conditions for grazing and adding-sliding bifurcations. In the present study, we will develop a new impact oscillator model at a micro scale, and environmental fluids will have significant effects on its dynamics, so an analytical solution will be derived to facilitate these analyses.

Building on the research background mentioned earlier, the technique involving magnetically actuated microrobots is primarily used for detecting cancer metastasis and facilitating drug delivery with blood vessels [28]. From a mechanical perspective, this topic is closely related to microrobot-fluid coupling, which involves the reciprocal interaction between a solid microrobot and the surrounding fluid in a two-phase flow system [29]. In such systems, the coupling relationship between the fluid’s flow dynamics and the movement of the microrobot plays an important role in the solid-liquid two-phase flow [30, 31]. Thus, methodologies for studying the dynamic responses of microrobots in biomedical applications typically involve direct experimental approach [8, 17, 19, 32] and numerical simulations [33] using commercial software, such as CFD [18], OpenFOAM [34], EDEM [35]. To simplify the interaction between the microrobot and fluid, our research proposes a simplified physical model where the microrobot moves in a fluid within a narrow cylindrical passage. The fluid’s viscosity is assumed constant, and the interaction between the blood cells and the microrobot is disregarded. This study formulates a dynamic model of a microrobot under external harmonic excitation to provide deeper insights into the mechanism of potential cancer metastasis. By analyzing the dynamic performance of the microrobot in fluid, we aim to develop analytical solutions for control strategies that could detect or prevent potential cancer metastasis.

The rest of the paper is organized as follows. In Sect "Physical model of the microrobot-vessel model" dynamic model of the microrobot-blood vessel interaction is formulated, and its analytical solution is obtained in Sect. "Physical model of the microrobot-vessel model". Analytical solution of the system is validated by the experimental data in Sect. "Experimental test and validation of the analytical". Transmitted force to the blood vessel wall and the resonance phenomenon of the microrobot-blood vessel interaction are analyzed in Sect. "Transmissibility of contact force and resonance behavior". In Sect. "Dynamic responses of the model under different parameters", the dynamic responses of the microrobot under the variation of related parameters are studied. Finally, conclusions are drawn in Sect. "Conclusions".

## Physical model of the microrobot-vessel model

**Fig. 2:**
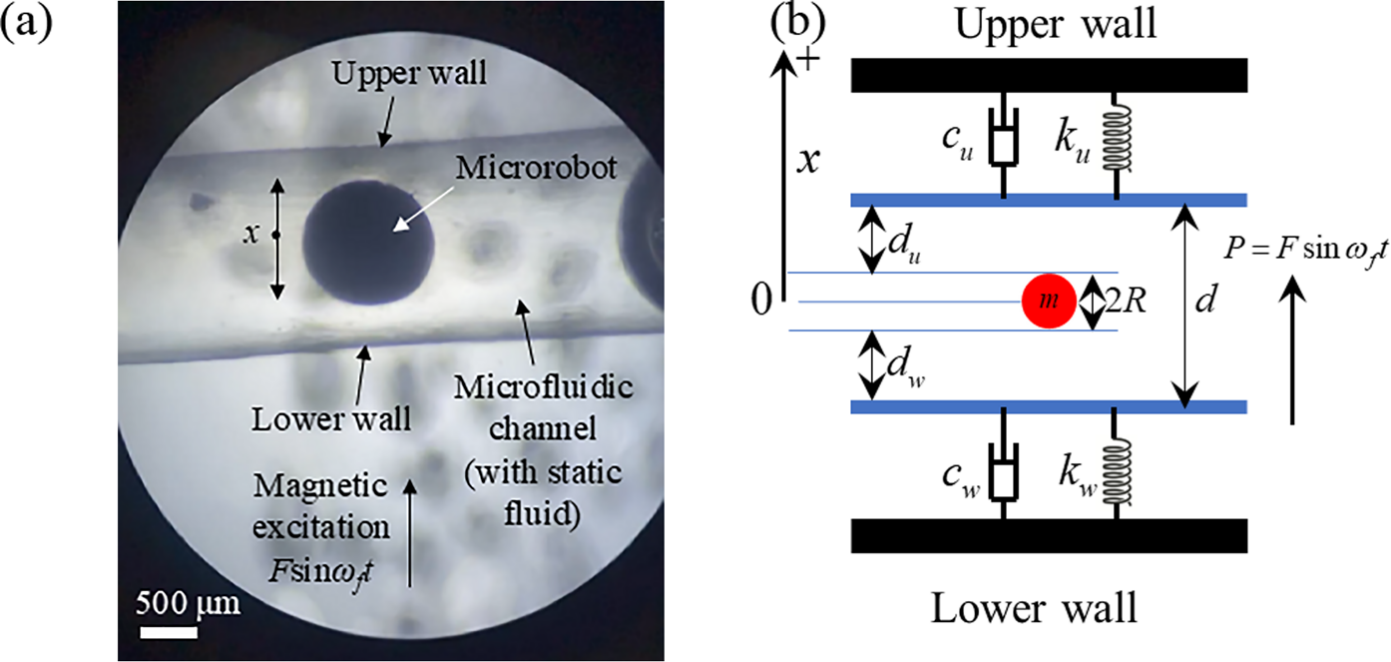
The microrobot-blood vessel interaction: **a** photograph of a microrobot in a microfluidic channel mimicking the blood vessel taken by a microscope; **b** one-dimensional physical model of a microrobot moving in the blood vessel under external harmonic excitation

In actual blood vessels, numerous cells move within the bloodstream and contact behavior exists among the cells, as well as between the cells and the blood vessel wall. A microrobot can be considered analogous to a cell moving within the vessel as shown in Fig. 2a. However, realistically analyzing the dynamics of a large number of cells, which effectively constitutes a vast wet granular system with intricate couplings between the microrobots, the fluid, and the flexible blood vessel wall, is challenging. Therefore, this section primarily introduces a physical model that simplifies the microrobot’s motion states within the blood vessel. To analyze the dynamic responses of the microrobot in the fluid, the complex interactions between the cells and the blood vessel wall are reduced to a one-dimensional dynamic model as shown in Fig. 2b, where a microrobot is excited vertically between upper and lower walls of the blood vessel. This section establishes the simplified physical model of the microrobot-blood vessel interaction, and subsequently formulates the dynamic model according to the equivalent physical model.

### Physical model of the microrobot-blood vessel model

The blood vessel walls, consisting of an upper and a lower wall, exhibit flexibility that is modelled using the spring-dashpot model. The microrobot, is treated as a particle in fluid, and is positioned at the center of the blood vessel, as illustrated in Fig. 2, where a notable distance separates the microrobot from the vessel walls. The harmonic excitation force $P=F\sin {\omega _f}t$, acting as an external force on the microrobot, is defined by a frequency ${\omega _f}$ and amplitude *F*. The vertical direction is designated as positive, and relevant physical parameters are detailed in Table 1. The initial conditions are set as *h*_0_ = 0 m and *v*_0_ = 0 m/s.

**Table 1:** Physical parameters of the microrobot-blood vessel model identified from experiment

Parameters	Values	Units
Mass of microrobot, *m*	2.50 × 10^−6^	kg
Radius of microrobot, *R*	5.50 × 10^−4^	m
Diameter of blood vessel, *d*	1.75 × 10^−3^	m
Upper microrobot-wall distance, *d*_*u*_	3.25 × 10^−4^	m
Lower microrobot-wall distance, *d*_*w*_	3.25 × 10^−4^	m
Stiffness coefficient of the upper wall, *k*_*u*_	10	N/m
Stiffness coefficient of the lower wall, *k*_*w*_	10	N/m
Damping coefficient of the upper wall, *c*_*u*_	6.0 × 10^−4^	N·s/m
Damping coefficient of the lower wall, *c*_*w*_	6.0 × 10^−4^	N·s/m
Damping coefficient of the fluid, *c*	5.0 × 10^−4^	N·s/m
Amplitude of the excitation force, *F*	1 × 10^−4^	N
Frequency of the excitation force, *ω*_*f*_	60	Hz

### Equations of motion of the microrobot-blood vessel model

Figure 2 depicts the motion of the microrobot within the blood vessel, which is analogized to the theoretical physical model of a particle moving in a fluid. In this vibrational system, there are three distinct states of motion: *contact between the particle and the upper wall*, *free motion*, and *contact between the particle and the lower wall*. The blood vessel wall is modelled as a linear spring-dashpot system characterized by constant stiffness and damping coefficients. Similarly, the fluid’s viscosity is represented by a constant value. This section formulates the dynamic model of these three motion states, drawing on principles from vibration theory.

*Contact between the particle and the upper wall* When the particle contacts with the upper wall, the equation of motion of the system is expressed as

1 $$ m\ddot{x}+\left({c}_{u}+c\right)\dot{x}+{k}_{u}\left(x-{d}_{u}\right)=F\text{sin}{\omega }_{f}t\; (t>{d}_{u})$$

*Free motion* When the particle has no contact with either wall, the equation of motion of the system is given as.

2 $$m\ddot{x}+c\dot{x}=F\text{sin}{\omega }_{f}t\; (-{d}_{w}<t<{d}_{u})$$

*Contact between the particle and the lower wall* When the particle contacts with the lower wall, the equation of motion of the system is written as.

3 $$m\ddot{x}+\left({c}_{w}+c\right)\dot{x}+{k}_{w}\left(x+{d}_{w}\right)=F\text{sin}{\omega}_{f}t\; (t<-{d}_{w})$$

## Analytical solution

This section solves the dynamic model of the microrobot-blood vessel interaction based on vibration theory. The model features damping and stiffness coefficients that are defined by constant values. Given these constants, analytical solutions for each of the three distinct motion states of this system can be derived.

When the particle contacts with the upper wall, the analytical solution is comprised of a transient solution ${x_{hu}}$ and a steady state solution ${x_{pu}}$. The transient solution is given by

4 $${x_{hu}}={X_u}{e^{ - {\xi _u}{\omega _{nu}}t}}\sin ({\omega _{du}}t+{\phi _u})$$

where the *X*_*u*_ is the amplitude value, ${\phi _u}$is the phase angle, the natural frequency is ${\omega _{nu}}=\sqrt {\frac{{{k_u}}}{m}}$, the damping ratio is ${\xi _u}=\frac{{c+{c_u}}}{{2 m{\omega _{nu}}}}=\frac{{c+{c_u}}}{{2\sqrt {m{k_u}} }}$, and the frequency of this system is ${\omega _{du}}={\omega _{nu}}\sqrt {1 - \xi _{u}^{2}}$.

The steady state solution is expressed as

5 $${x_{pu}}={A_u}{\beta _u}\sin \left( {{\omega _f}t - {\varphi _u}} \right)+{d_u}$$

where the frequency ratio is expressed as ${r_u}=\frac{{{\omega _f}}}{{{\omega _{nu}}}}$, the amplitude is ${A_u}=\frac{F}{{{k_u}}}$, the magnification factor is ${\beta _u}=\frac{1}{{\sqrt {{{\left( {1 - r_{u}^{2}} \right)}^2}+{{(2{r_u}{\xi _u})}^2}} }}$, and the phase angle is ${\varphi _u}={\tan ^{ - 1}}\left( {\frac{{2{r_u}{\xi _u}}}{{1 - r_{u}^{2}}}} \right)$

The complete solution can be written as

6 $${x_u}={x_{hu}}+{x_{pu}}={X_u}{e^{ - {\xi _u}{\omega _{nu}}t}}\sin ({\omega _{du}}t+{\phi _u})+{A_u}{\beta _u}\sin \left( {{\omega _f}t - {\varphi _u}} \right)+{d_u}$$

The motion of the microrobot involves continuously transitioning between the three distinct motion states in the fluid. The amplitude *X*_*u*_ and phase angle ${\phi _u}$ corresponding to the complete solution can be solved at the arbitrary time *τ*. Therefore, the initial conditions are assumed as $t=\tau, x\left(\tau\; \right)={h}_{0},\dot{x}\left(\tau\right)={v}_{0}$, namely, the amplitude *X*_*u*_ and phase angle ${\phi _u}$ can be solved according to the initial conditions.

To solve the amplitude and phase angle, the velocity of the microrobot can be acquired by differentiating the displacement with respect to time as

7 $$ \dot{x}_{u}  = X_{u} e^{{ - \xi _{u} \omega _{{nu}} t}} \left[ { - \xi _{u} \omega _{{nu}} {\text{sin}}(\omega _{{du}} t + \varphi _{u} ) + \omega _{{du}} {\text{cos}}(\omega _{{du}} t + \varphi _{u} )} \right] + \omega _{f} A_{u} \beta _{u} {\text{cos}}\left( {\omega _{f} t - \phi _{u} } \right) $$

Substituting Eqs. (6) and (7) into the initial conditions,

8 $$\left\{\begin{array}{c}{x}_{u}={X}_{u}{e}^{-{\xi}_{u}{\omega}_{nu}\tau}\text{sin}({\omega}_{du}\tau\;+{\varphi}_{u})+{A}_{u}{\beta}_{u}\text{sin}\left({\omega}_{f}\tau-{\phi}_{u}\right)-{d}_{u}={h}_{0}\\ \;{\dot{x}}_{u}={X}_{u}{e}^{-{\xi}_{u}{\omega}_{nu}\tau}\left[-{\xi}_{u}{\omega}_{nu}\text{sin}({\omega}_{du}\tau+{\varphi}_{u})+{\omega}_{du}\text{cos}({\omega}_{du}\tau+{\varphi}_{u})\right]+{\omega}_{f}{A}_{u}{\beta}_{u}\text{cos}\left({\omega}_{f}\tau-{\phi}_{u}\right)={v}_{0}\end{array}\right.$$

According to Eq. (8), the amplitude *X*_*u*_ and phase angle ${\phi _u}$can be obtained as

$$\left\{ \begin{gathered} {X_u}= - \frac{{\sqrt {G_{u}^{2}+H_{u}^{2}{\mkern 1mu} \omega _{{du}}^{2}} }}{{{\omega _{du}}{e^{ - {\xi _u}{\omega _{nu}}\tau }}}} \hfill \\ {\phi _u}= - 2{\mkern 1mu} {\text{atan}}\left( {\frac{{{G_u}+\sqrt {G_{u}^{2}+H_{u}^{2}{\mkern 1mu} \omega _{{du}}^{2}} }}{{{H_u}{\mkern 1mu} {\omega _{du}}}}} \right) - {\omega _{du}}{\mkern 1mu} \tau \hfill \\ \end{gathered} \right.$$,

9 $$\left\{ \begin{gathered} {H_u}={h_0} - {A_u}{\beta _u}\sin \left( {{\omega _f}\tau - {\varphi _u}} \right) - {d_u} \hfill \\ {G_u}={v_0}+{H_u}{\xi _u}{\omega _{nu}} - {\omega _f}{A_u}{\beta _u}\cos \left( {{\omega _f}\tau - {\varphi _u}} \right) \hfill \\ \end{gathered} \right.$$

When the microrobot has no contact with the wall, the solution of the equation of motion in Eq. (2), can be solved as the transient solution ${x_{{h_0}}}$ and steady state solution${x_{{p_0}}}$. The transient solution can be written as

10 $${x_{{h_0}}}={B_1}+{B_2}{e^{\left( {\frac{{ - ct}}{m}} \right)}}$$

where *B*_1_ and *B*_2_ are the unknown coefficients.

The steady state solution can be expressed as

11 $${x_{{p_0}}}={A_0}\sin ({\omega _f}t - \theta )$$

where the amplitude *A*_0_ and phase angle *θ* can be written as

12 $$\left\{ \begin{gathered} \theta = - arc\cos \left( {\frac{{m{\omega _f}}}{{\sqrt {{c^2}+{m^2}\omega _{f}^{2}} }}} \right) \hfill \\ {X_0}=\frac{F}{{\left( {c{\omega _f}\sin \theta - m\omega _{f}^{2}\cos \theta } \right)}} \hfill \\ \end{gathered} \right.$$

The complete solution for the free motion can be written as

13 $${x_0}={x_{{h_0}}}+{x_{{p_0}}}={B_1}+{B_2}{e^{\left( {\frac{{ - ct}}{m}} \right)}}+{A_0}\sin ({\omega _f}t - \theta )$$

Therefore, the velocity of the microrobot freely moves in the fluid is expressed as

14 $$ {\dot{x}}_{0}=-\frac{{B}_{2}c}{m}{e}^{\left(\frac{-ct}{m}\right)}+{A}_{0}{\omega}_{f}\text{cos}({\omega}_{f}t-\theta)$$

Substituting Eqs. (13) and  (14) into the initial conditions $t=\tau\;,x\left(\tau\; \right)={h}_{0},\dot{x}\left(\tau\;\right)={v}_{0}$

15 $$\left\{ \begin{gathered} {h_0}={B_1}+{B_2}{e^{\left( {\frac{{ - c\tau }}{m}} \right)}}+{A_0}\sin ({\omega _f}\tau - \theta ) \hfill \\ {v_0}= - \frac{{{B_2}c}}{m}{e^{\left( {\frac{{ - c\tau }}{m}} \right)}}+{A_0}{\omega _f}\cos ({\omega _f}\tau - \theta ) \hfill \\ \end{gathered} \right.$$

Accordingly, two unknown coefficients can be solved as

16 $$\left\{ \begin{gathered}   B_{2}  =  - \frac{m}{{ce^{{\left( {\frac{{ - c\tau }}{m}} \right)}} }}\left[ {v_{0}  - A_{0} \omega _{f} \cos (\omega _{f} \tau  - \theta )} \right] \hfill \\   B_{1}  = h_{0}  - A_{0} \sin (\omega _{f} \tau  - \theta ) - B_{2} e^{{\left( {\frac{{ - c\tau }}{m}} \right)}} \;\;\;\;\;\; \hfill \\  \end{gathered}  \right. $$

When the particle contacts with the lower wall, the solution of the equation of motion in Eq. (3), includes the transient solution ${x_{hw}}$ and steady state solution ${x_{pw}}$. The transient solution is given by

17 $${x_{hw}}={X_w}{e^{ - {\xi _w}{\omega _{nw}}t}}\sin ({\omega _{dw}}t+{\phi _w})$$

where the *w*_*u*_ is the amplitude value, ${\phi _w}$is the phase angle, the natural frequency is ${\omega _{nw}}=\sqrt {\frac{{{k_w}}}{m}}$, the damping ratio is ${\xi _w}=\frac{{c+{c_w}}}{{2 m{\omega _{nw}}}}=\frac{{c+{c_w}}}{{2\sqrt {m{k_w}} }}$, the frequency of this system is ${\omega _{dw}}={\omega _{nw}}\sqrt {1 - \xi _{w}^{2}}$.

The steady state solution is expressed as

18 $${x_{pw}}={A_w}{\beta _w}\sin \left( {{\omega _f}t - {\varphi _w}} \right)+{d_w}$$

where the frequency ratio is expressed as ${r_w}=\frac{{{\omega _f}}}{{{\omega _{nw}}}}$, the amplitude is ${A_w}=\frac{F}{{{k_w}}}$, the magnification factor is ${\beta _w}=\frac{1}{{\sqrt {{{\left( {1 - r_{w}^{2}} \right)}^2}+{{(2{r_w}{\xi _w})}^2}} }}$, the phase angle is ${\varphi _u}={\tan ^{ - 1}}\left( {\frac{{2{r_w}{\xi _w}}}{{1 - r_{w}^{2}}}} \right)$

The complete solution can be written as

19 $${x_w}={x_{hw}}+{x_{pw}}={X_w}{e^{ - {\xi _w}{\omega _{nw}}t}}\sin ({\omega _{dw}}t+{\phi _w})+{A_w}{\beta _w}\sin \left( {{\omega _f}t - {\varphi _w}} \right) - {d_w}$$

The velocity of the particle can be acquired by differential the displacement with respect to time

20 $${\dot{x}}_{w}={X}_{w}{e}^{-{\xi}_{w}{\omega}_{nw}t}\left[-{\xi}_{w}{\omega}_{nw}\text{sin}({\omega}_{dw}t+{\varphi}_{w})+{\omega}_{dw}\text{cos}({\omega}_{dw}t+{\varphi}_{w})\right]+{\omega}_{f}{A}_{w}{\beta}_{w}\text{cos}\left({\omega}_{f}t-{\phi}_{w}\right)$$

Substituting Eqs. (19) and (20) into the initial conditions $t=\tau\;,x\left(\tau\;\right)={h}_{0},\dot{x}\left(\tau\;\right)={v}_{0}$

21 $$\left\{\begin{array}{c}{x}_{w}={X}_{w}{e}^{-{\xi}_{w}{\omega}_{nw}\tau}\text{sin}({\omega}_{dw}\tau +{\varphi }_{w})+{A}_{w}{\beta }_{w}\text{sin}\left({\omega  }_{f}\tau -{\phi }_{w}\right)-{d}_{w}={h}_{0}\\ \;{\dot{x}}_{w}={X}_{w}{e}^{-{\xi }_{w}{\omega }_{nw}\tau}\left[-{\xi}_{w}{\omega}_{nw}\text{sin}({\omega}_{dw}\tau+{\varphi }_{w})+{\omega}_{dw}\text{cos}({\omega}_{dw}\tau+{\varphi}_{w})\right]+{\omega}_{f}{A}_{w}{\beta}_{w}\text{cos}\left({\omega}_{f}\tau-{\phi}_{w}\right)={v}_{0}\end{array}\right.$$

According to Eq. (21), the amplitude *X*_*w*_ and phase angle ${\phi _w}$can be obtained as

$$\left\{ \begin{gathered} {X_w}= - \frac{{\sqrt {G_{w}^{2}+H_{w}^{2}{\mkern 1mu} \omega _{{dw}}^{2}} }}{{{\omega _{dw}}{e^{ - {\xi _w}{\omega _{nw}}\tau }}}} \hfill \\ {\phi _w}= - 2{\mkern 1mu} {\text{atan}}\left( {\frac{{{G_w}+\sqrt {G_{w}^{2}+H_{w}^{2}{\mkern 1mu} \omega _{{dw}}^{2}} }}{{{H_w}{\mkern 1mu} {\omega _{dw}}}}} \right) - {\omega _{dw}}{\mkern 1mu} \tau \hfill \\ \end{gathered} \right.$$,

22 $$ \left\{ \begin{gathered}   H_{w}  = h_{0}  - A_{w} \beta _{w} \sin \left( {\omega _{f} \tau  - \varphi _{w} } \right) + d_{w} \;\;\;\;\;\;\;\;\;\;\;\;\; \hfill \\   G_{w}  = v_{0}  + H_{w} \xi _{w} \omega _{{nw}}  - \omega _{f} A_{w} \beta _{w} \cos \left( {\omega _{f} \tau  - \varphi _{w} } \right) \hfill \\  \end{gathered}  \right. $$

## Experimental test and validation of the analytical solutions

To verify the correctness of the analytical solutions, this section conducts a specific experimental test aligned with the theoretical model shown in Fig. 2b. The analytical solutions in Sect. "Physical model of the microrobot-vessel model", which include the displacement and velocity of the microrobot, are validated against the experimental data in this section.

**Fig. 3:**
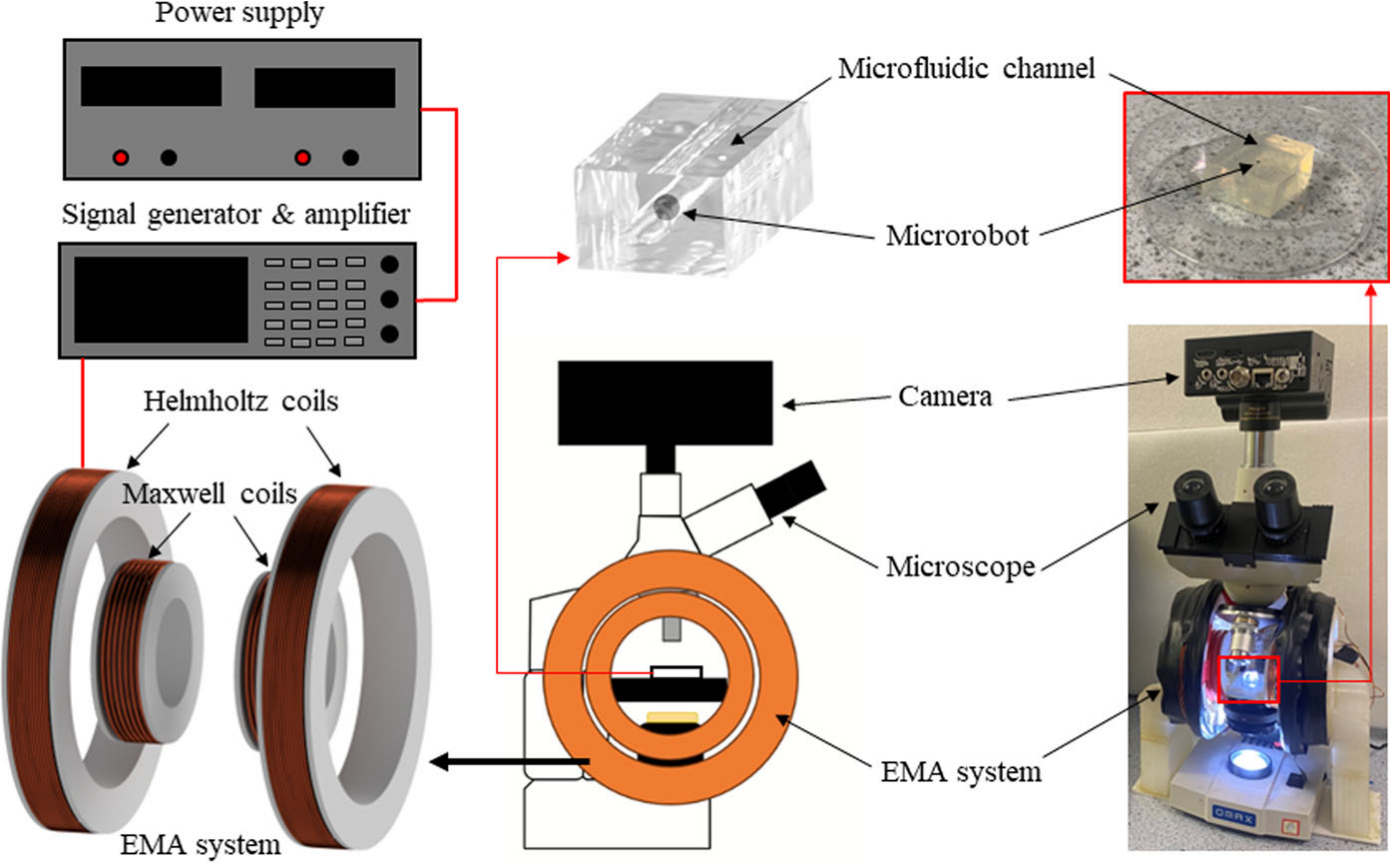
Schematic diagram of the experimental setup, which consists of an electromagnetic actuation (EMA) system fitted to a microscope controlled by a signal generator connected to a power supply and amplifier. The particle motion was captured on a high-speed camera through the imaging port of the microscope

### Experiment rig

The experimental rig shown in Fig. 3 consists of an electromagnetic actuation system containing a pair of Helmholtz coils to produce a uniform magnetic field and a pair of Maxwell coils to produce a magnetic field gradient. The Helmholtz coils measured 150 cm across the diameter and consisted of 250 turns in each coil, the Maxwell coils measured 7 cm across the diameter and consisted of 280 turns in each coil producing a maximum uniform and gradient field of 5 mT and 1.5 T/m respectively. To fabricate the microfluidic channels circular ABS plastic of 1.75 mm cross section was used as a scaffold for the channel whilst agar of different mixture percentages with water was poured into petri dishes and left to set for 1 h, once set, the ABS was carefully removed leaving behind a circular cross-sectional channel of 1.75 mm diameter. A total of 6 samples were made, ranging from 0.6 to 1.6% wt agar in water. Each agar sample was placed in the center of the electromagnetic actuation system, such that the channel was directed perpendicular to the direction of the magnetic fields. De ionized water was injected into the channel along with the particle. For each round of sample testing, the particle was subjected to sinusoidal forcing at frequencies ranging from 5 Hz to 70 Hz. A high speed camera (Chronos 2.1, Kron Technologies, Canada) recording at 2000 frames per second captured the particle motion, video files were exported to a tracking software (Tracker software) to track the dynamics of the particle at each frequency.

To fabricate the microparticles, PDMS elastomer (Slygard 184, Dow Corning, USA) base and curing agents were mixed in the standard 10:1 ratio by weight. The ratio was chosen because of the curing profile and elastic properties. The NdFeB magnetic powder (Neo Magnequench, Singapore) was then mixed into the uncured elastomer at a ratio of 2:1 particle elastomer by weight and stirred for 15 min to ensure an even distribution of magnetic particles within the elastomer. The newly mixed elastomer-particle mixture was degassed in a vacuum oven for 30 min to remove air, followed by heating for 15 min at 70 °C to partially cure and subsequently increase the viscosity of the mixture. The aqueous solution consisted of 0.5 ml Triton X-100 surfactant mixed with 25 ml deionized water. 1 ml of the elastomer-particle mixture was added dropwise to the aqueous solution using a pipette and stirred at 1000 rpm using an overhead mechanical stirrer and heated at 70 °C for 2.5 h using a hotplate. The prepared magnetic microparticles were collected, washed with deionized water, and left to dry at room temperature. The magnetic particle used in the experiment was selected from the dry batch.

To fabricate the microfluidic channels circular ABS plastic of 1.75 mm cross section was used as a scaffold for the channel whilst agar of different mixture percentages with water was poured into petri dishes and left to set for 1 h, once set, the ABS was carefully removed leaving behind a circular cross-sectional channel of 1.75 mm diameter.

### The comparison between experimental data and analytical solution

The relevant parameters depicted in Fig. 2b are listed in Table 1. The microrobot in fluid exhibits three distinct motion states, with transitions between these states determined by the initial conditions at any given moment. Consequently, the amplitude and phase angle of the transient solution should be calculated at arbitrary moments, not just at the initial moment (*t* = 0), to ensure the detection of any contact between the microrobot and the blood vessel wall in analytical solutions.

**Fig. 4:**
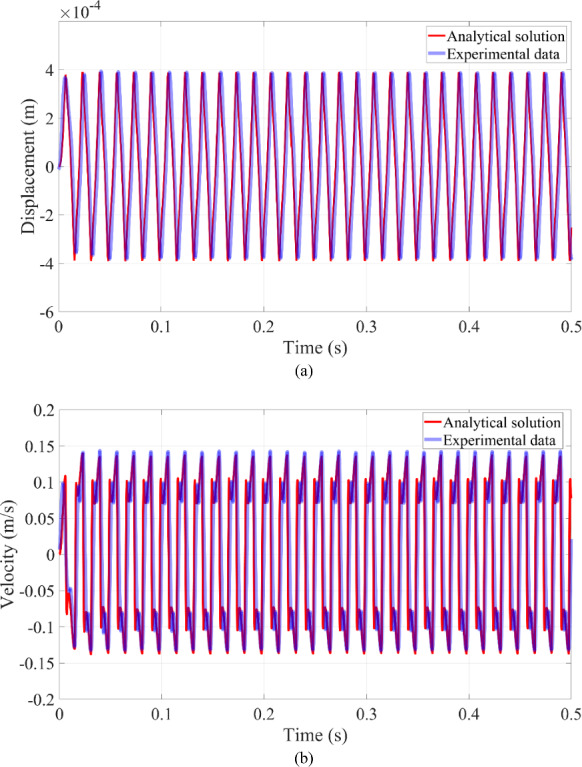
The dynamic response of the microrobot in fluid: **a** displacement and **b** velocity of the microrobot

The displacement of the microrobot, as shown in Fig. 4a, aligns with the experimental data, and the velocity of the microrobot remains consistent with the experimental results in Fig. 4b. As can be seen from the figure, the dynamic responses of the microrobot stabilize after only a few cycles in fluid. The contact behavior between the microrobot and blood vessel wall is clearly evident in the velocity profile of the microrobot in Fig. 4b, where the velocity first decreases and then increases under harmonic excitation when contact occurs. However, discrepancies remain between the experimental data and analytical solutions. These discrepancies are primarily due to the experimental setup’s inability to ensure that the microrobot moves strictly in one direction in fluid. Despite these discrepancies, the differences do not impact the validity of the analytical solutions. Thus, the analytical solutions are confirmed by the experimental data and serve as a reliable reference for analyzing the dynamic responses of the system as related parameters vary.

## Transmissibility of contact force and resonance behavior

This section explains the propagation of the contact force when the particle contacts the blood vessel wall. The transmission law of the contact force between the particle and blood vessel wall plays an important role in the detection process of potential cancer metastasis by microrobot [36, 37]. On the one hand, the investigation of transmission law of the contact force on the blood vessel wall can provide a theoretical guidance for the control strategy of the microrobot. On the other hand, the magnitude of the contact force plays a decisive role in cancer metastasis detection using a microrobot because the larger contact force between the microrobot and blood vessel wall may puncture the blood vessel wall so as to damage human tissues [38, 39]. It is necessary to study the transmissibility of the contact force caused by the impact behavior between the microrobot and the blood vessel wall. Furthermore, when the microrobot is in constant contact with the blood vessel wall, the resonance phenomenon has a significant effect on the transmission of the contact force as well, hence, this section provides the dynamic response of the resonance behavior in this system.

### Contact force transmission

The transmission phenomenon in vibration systems refers to the process by which vibrational energy travels through the blood vessel wall. This phenomenon occurs because vibrations are essentially mechanical waves that propagate through different media, carrying energy from one location to another. When a microrobot impacts with blood vessel wall, the kinetic energy is transferred to the strain energy stored in the blood vessel wall, and the stored strain energy is applied to the vibration of the microrobot, creating a wave that travels in the blood vessel wall. This phenomenon is influenced by material properties, boundary conditions, and resonance effects. This understanding is crucial in fields like engineering, where controlling vibration transmission is often necessary to ensure the stability of the microrobot.

Although the contact behaviors may happen either between the microrobot and the upper blood vessel wall or between the microrobot and the lower blood vessel wall, the transmission law of the contact force in the blood vessel wall is the same for both. Therefore, to this end, the contact behavior between the microrobot and the upper blood vessel wall is selected to analyze the contact force transmission. The force transmitted to the blood vessel wall in the steady state solution can be expressed as

23 $$  {F}_{T}={k}_{u}\left({x}_{pu}-{d}_{u}\right)+{c}_{u}{\dot{x}}_{pu}$$

Based on the steady state solution in Eq. (5), differentiating Eq. (5) with respect to time, the ${\dot{x}}_{pu}$is expressed as

24 $$ {\dot{x}}_{pu}={\omega }_{f}{A}_{u}{\beta }_{u}\cos\left({\omega }_{f}t-{\phi }_{u}\right)$$

Substituting Eqs. (5) and  (23) into Eq. (23), the contact force transmission is given by

25 $$  {F}_{T}={k}_{u}\left({x}_{pu}-{d}_{u}\right)+{c}_{u}{\dot{x}}_{pu}={k}_{u}{A}_{u}{\beta }_{u}\text{sin}\left({\omega }_{f}t-{\phi }_{u}\right)+{c}_{u}{\omega  }_{f}{A}_{u}{\beta }_{u}\text{cos}\left({\omega  }_{f}t-{\phi  }_{u}\right) \;=\;{k}_{u}{A}_{u}{\beta}_{u}\text{sin}\left({\omega}_{f}t-{\phi}_{u}\right)+{c}_{u}{\omega}_{f}{A}_{u}{\beta}_{u}\text{cos}\left({\omega}_{f}t-{\phi}_{u}\right)$$

$$ = A_{u} \beta   _{u} \sqrt {k_{u}^{2} + \left( {c_{u} \omega _{f} } \right)^{2} } {\sin}\left( {\omega _{f} t - \overline{\phi } } \right)$$

where $$\bar {\varphi }={\varphi _u} - {\varphi _t},{\varphi _t}={\tan ^{ - 1}}\left( {\frac{{{c_u}{\omega _f}}}{{{k_u}}}} \right)={\tan ^{ - 1}}\left( {2{r_u}{\xi _u}} \right)$$.

Since ${A_u}={F \mathord{\left/ {\vphantom {F {{k_u}}}} \right. \kern-0pt} {{k_u}}}$, the contact force transmission can be rewritten by means of the amplitude of the excitation force

26 $$\begin{gathered} {F_T}={A_u}{\beta _u}\sqrt {k_{u}^{2}+{{\left( {{c_u}{\omega _f}} \right)}^2}} \sin \left( {{\omega _f}t - \bar {\varphi }} \right) \hfill \\ =F{\beta _u}\sqrt {1+{{\left( {2{r_u}{\xi _u}} \right)}^2}} \sin \left( {{\omega _f}t - \bar {\varphi }} \right) \hfill \\ =F{\beta _T}\sin \left( {{\omega _f}t - \bar {\varphi }} \right) \hfill \\ \end{gathered}$$

The coefficient ${\beta _T}$represents the ratio between the amplitude of the transmitted force and the amplitude of the excitation force, which is referred to as the transmissibility that is expressed as

27 $${\beta _T}={\beta _u}\sqrt {1+{{\left( {2{r_u}{\xi _u}} \right)}^2}} =\frac{{\sqrt {1+{{\left( {2{r_u}{\xi _u}} \right)}^2}} }}{{\sqrt {{{\left( {1 - r_{u}^{2}} \right)}^2}+{{\left( {2{r_u}{\xi _u}} \right)}^2}} }}$$

**Fig. 5:**
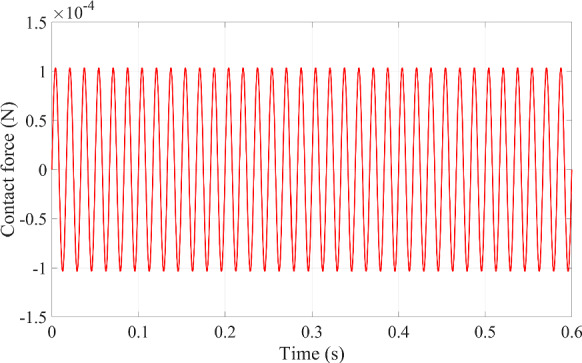
The contact force caused by the impact behavior between the microrobot and the blood vessel wall

**Fig. 6:**
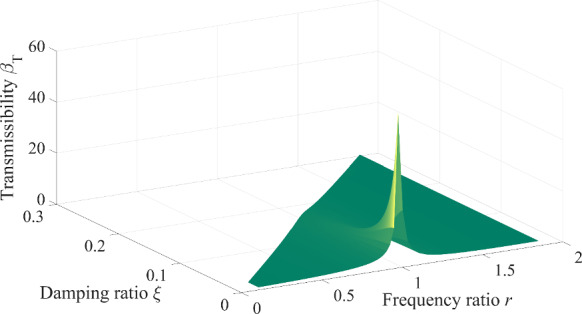
Transmissibility relating the damping ratio and frequency ratio of the system

**Fig. 7:**
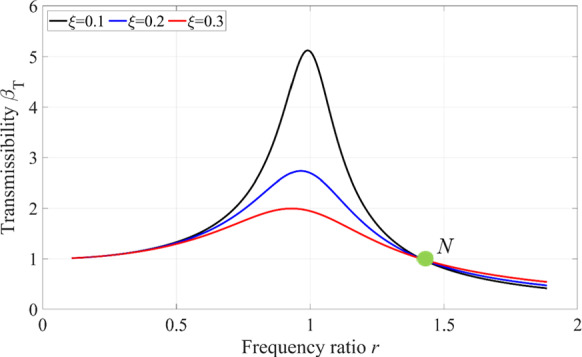
Transmissibility of the vibration system

When the contact behavior between the microrobot and blood vessel wall happens, the contact force has a stable transmission to the blood vessel wall under harmonic excitation in Fig. 5. The maximum contact force transmitted by the external excitation is close to the magnitude of the excitation force 1 × 10^−4^ N. Nonetheless, it is important to carefully observe that the maximum contact force is slightly greater than the excitation force because the transmissibility is slightly greater than one, as shown in Figs. 6 and 7.

Further, the magnitude of the amplitude of the contact force is controlled by the transmissibility and excitation force in Eq. (26). Since the magnitude of the external excitation force is determined in advance, the internal reason emanates from the transmissibility that is the function of the damping ratio and frequency ratio in Eq. (27). Therefore, the transmitted contact force is closely related to the frequency ratio and damping ratio in Eq. (27). The relationship between the transmissibility, frequency ratio and damping ratio can be seen in Fig. 6. The transmissibility increases with the decreasing damping ratio, its maximum value is obtained when the frequency ratio is equal to one. However, when the entire system is confirmed, the damping ratio of the system is determined in advance, and the frequency ratio has a significant effect on the transmissibility, as depicted in Fig. 7. When the frequency ratio is larger than $\sqrt 2$ (this value can be obtained from Eq. (27)) at the point *N* in Fig. 7, the transmissibility is smaller than one no matter what the damping ratio is, the amplitude of the transmitted contact force is less than the amplitude of the external excitation force. The amplitude of the transmitted contact force can be increased by increasing the damping ratio. On the contrary, when the frequency ratio is less than $\sqrt 2$, the transmissibility is greater than one, which results in that the amplitude of the transmitted contact force is greater than the amplitude of the external excitation force. The amplitude of the transmitted contact force can be reduced by increasing the damping ratio.

### Resonance phenomenon

When the forcing frequency is equal to the natural frequency of this system, the frequency ratio is equal to one (*r*_*u*_=1). The phase angle is equal to ${\pi \mathord{\left/ {\vphantom {\pi 2}} \right. \kern-0pt} 2}$. The magnification factor of the steady state solution is reduced as

28 $${\beta _u}=\frac{1}{{\sqrt {{{\left( {1 - r_{u}^{2}} \right)}^2}+{{(2{r_u}{\xi _u})}^2}} }}\mathop \Rightarrow \limits^{{{r_u}=1}} {\beta _r}=\frac{1}{{2\xi }}$$

The steady state solution from the contact between the microrobot and upper blood vessel wall in Eq. (5) can be simplified as

29 $${x_{pu}}={A_u}{\beta _u}\sin \left( {{\omega _f}t - {\varphi _u}} \right)+{d_u} \Rightarrow {x_r}= - {A_u}{\beta _r}\cos {\omega _f}t+{d_u}$$

The velocity of the microrobot is expressed as

30 $$ {\dot{x}}_{r}={A}_{u}{\beta }_{r}{\omega }_{f}\text{sin}{\omega }_{f}t$$

It is worth noting that the resonance phenomenon only happens during impact between the microrobot and blood vessel wall. The free motion of the microrobot in the fluid is ignored in studying resonance behavior. Accordingly, the damping coefficient of the fluid is equal to zero (*c* = 0), the damping ratio is ${\xi _u}={{{c_u}} \mathord{\left/ {\vphantom {{{c_u}} {2 m{\omega _{nu}}}}} \right. \kern-0pt} {2 m{\omega _{nu}}}}$. It clearly knows that the magnification factor is infinite when the damping ratio is equal to zero. Nonetheless, the damping coefficient of blood vessel wall is never equal to zero, hence, the displacement of this system will not approach infinite as well. However, when the frequency ratio is calculated as $r=\sqrt {1 - 2{\xi ^2}}$, the maximum magnification factor is expressed as ${\beta _{\hbox{max} }}={1 \mathord{\left/ {\vphantom {1 {2\xi \sqrt {1 - {\xi ^2}} }}} \right. \kern-0pt} {2\xi \sqrt {1 - {\xi ^2}} }}$. Since the damping ratio of this system is very small, both the maximum displacement and velocity of the microrobot calculated by the maximum magnification have slight differences from the dynamic response corresponding to the resonance behavior. Moreover, from Eqs. (28)–(30), it can be clearly observed that the magnification factor primarily dominates the dynamic responses of the resonance phenomenon when the excitation force and frequency have been confirmed. Namely, the resonance phenomenon from the dynamic response of contact behavior between the microrobot and blood vessel wall is closely pertinent to the damping coefficients representing the physical properties of the blood vessel wall.

**Fig. 8:**
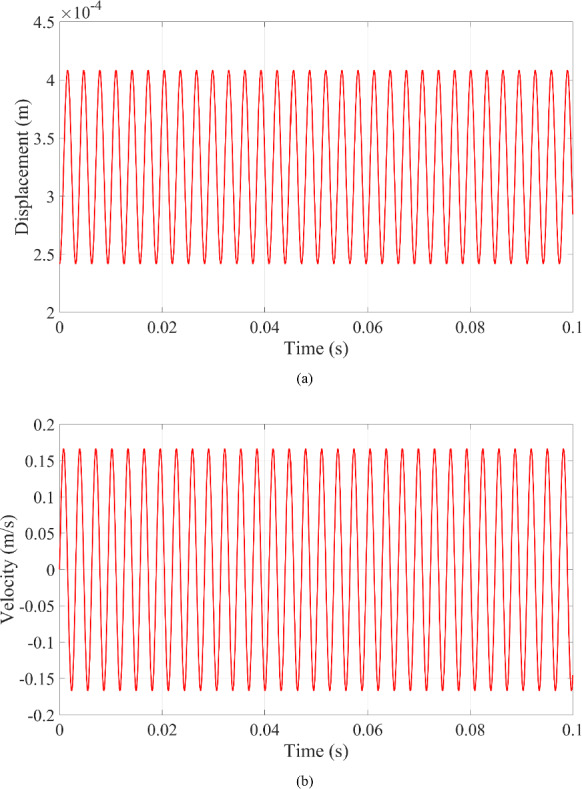
Dynamic responses of the microrobot with impact at resonance:** a** displacement of the microrobot;** b** velocity of the microrobot

When the contact behavior between the microrobot and blood vessel wall generates the resonance phenomenon, the dynamic responses in Eqs. (29) and (30), including the displacement in Fig. 8a, and the velocity in Fig. 8b, of the microrobot has a stable vibration status with a constant amplitude. In other words, the resonance phenomenon makes the microrobot vibrate back and forth against the blood vessel wall in Fig. 8.

**Fig. 9:**
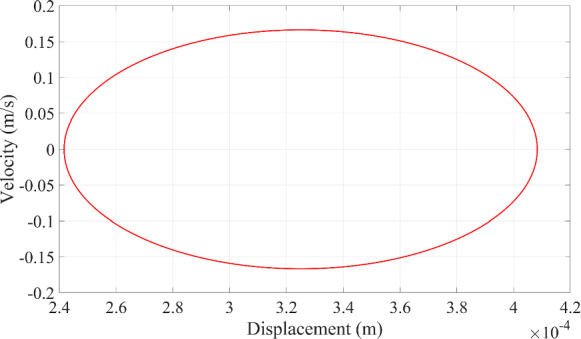
The relationship between the displacement and velocity of the microrobot at resonance

Since the resonance phenomenon keeps a periodic motion in Fig. 8, the stability of the vibration of the microrobot can be seen in Fig. 9 as well. Since the dynamic response of the microrobot at the resonance phenomenon is mainly controlled by the magnification factor that is the function of the damping ratio in Eq. (28), the prediction of the dynamic responses of the microrobot is implemented when the damping ratio has these different values (0.01, 0.03, 0.05, 0.07, 0.09).

**Fig. 10:**
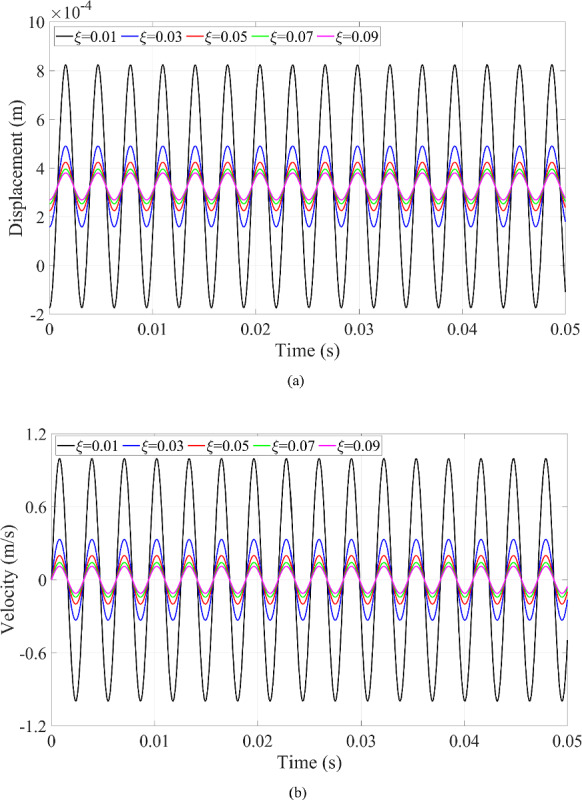
Dynamic responses of the microrobot at resonance with the variation of damping ratio: **a** displacement of the microrobot; **b** velocity of the microrobot

**Fig. 11:**
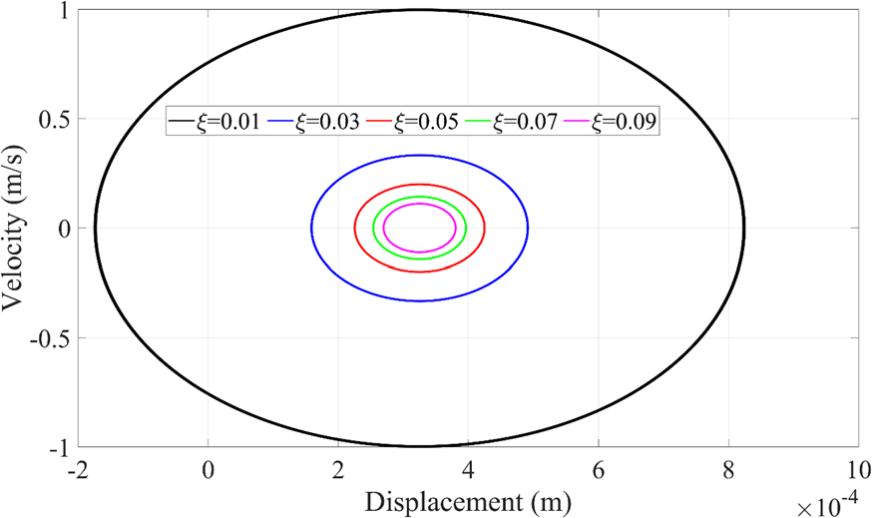
The relationship between the displacement and velocity of the microrobot with the variation of the damping ratio of the system

The displacement in Fig. 10a and velocity in Fig. 10b of the microrobot maintain the stable properties of the resonance phenomenon when the damping ratio is changed. Furthermore, the amplitude of the dynamic responses increases with the decreasing damping ratio because the smaller damping ratio dissipates less kinetic energy of the microrobot. Namely, the remaining kinetic energy of the microrobot, which is greater with a smaller damping ratio, contributes to the impact behavior, leading to its displacement and velocity having larger amplitudes compared to those with a larger damping ratio. That is why the area of the circle from the relationship between displacement and velocity displayed in Fig. 11 increases with the reduced damping ratio.

## Dynamic responses of the model under different parameters

The correctness of the analytical solutions of the system shown in Fig. 2b has been proved in Sect. "Experimental test and validation of the analytical". Therefore, the analytical solutions can serve as the reference to measure the effect of the related parameters on the dynamic responses of the microrobot in the fluid, which provides a series of rich simulated results and a theoretical foundation for the control strategy design of the microrobot. Since the related parameters include the excitation frequency and force, the damping coefficient of the fluid, and the damping and stiffness coefficient of the blood vessel wall, the effects of these parameters on the dynamic responses of the microrobot are tested in this section, respectively. The simulated parameters are displayed in Table 1.

**Fig. 12:**
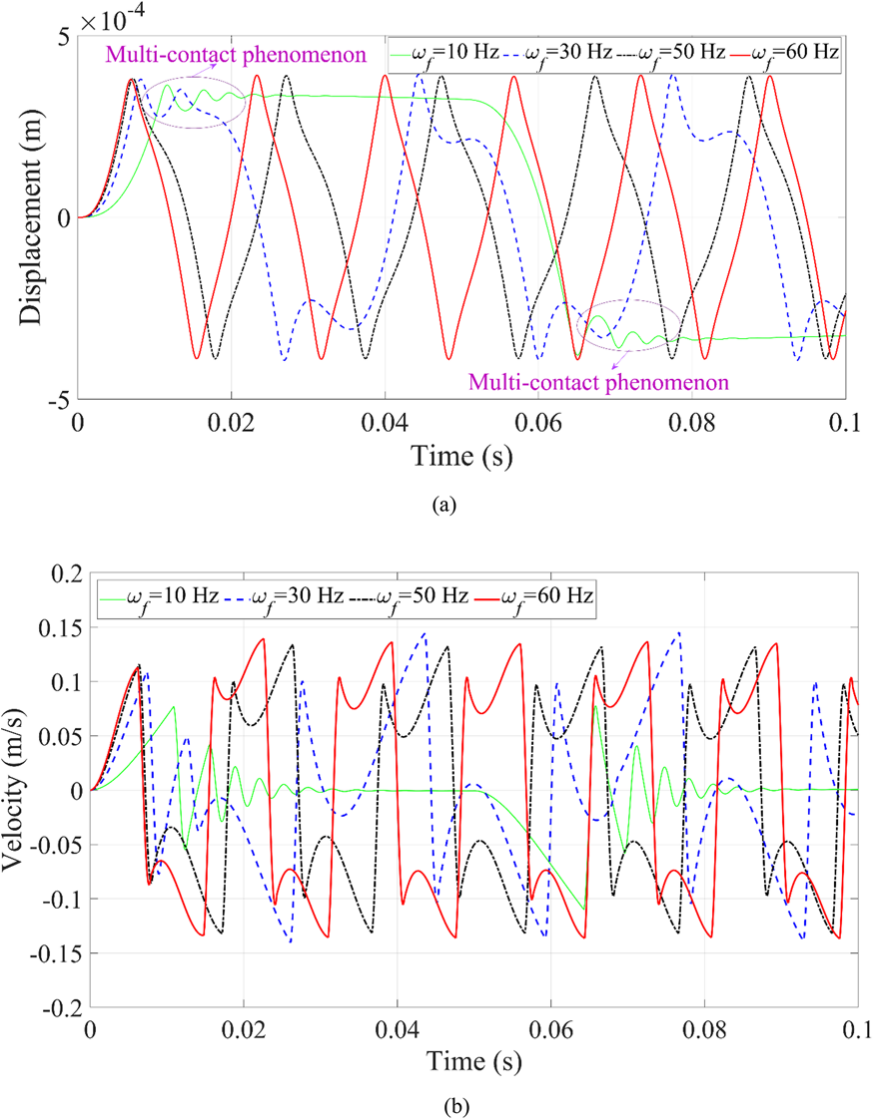
Dynamic responses of the microrobot under the variation of excitation frequency: **a** displacement of the microrobot; **b** velocity of the microrobot

**Fig. 13:**
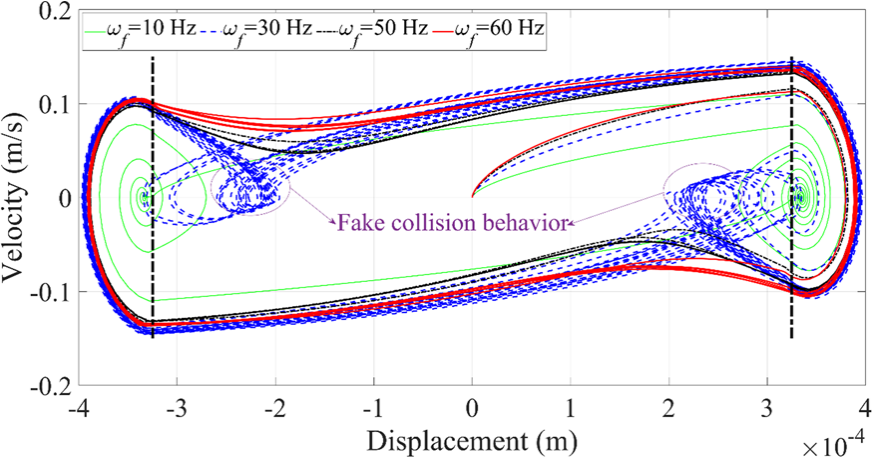
Phase trajectories of the microrobot under the variation of the excitation frequency

The first test scenario involves different excitation frequencies, and the dynamic responses of the microrobot can be seen in Figs. 12 and 13. When the excitation frequency is reduced, the entire motion duration of the microrobot moving from the upper to the lower blood vessel wall becomes longer, and the feedback becomes slower with a low frequency. At an excitation frequency of 10 Hz, the microrobot exhibits a multi-contact phenomenon with the upper blood vessel wall prior to entering free motion states, as shown in Fig. 12a. Likewise, this multi-contact phenomenon also occurs with the lower blood vessel wall. This results in the microrobot displaying characteristics of multi-periodic motion. The displacement shows multiple contacts with the upper blood vessel wall from around 0.01–0.03 s, and multiple vibrations with the lower blood vessel wall from around 0.065 s to 0.082 s. Moreover, when multi-contact behavior happens, the microrobot pauses at either the upper or lower blood vessel wall for a while after contact, as depicted in Fig. 12a.

The velocity of the microrobot shows zero immediately after these contacts, as illustrated in Fig. 12b. The muti-contact behavior also occurs at an excitation frequency of 30 Hz. However, at 50 Hz or higher, the multi-contact phenomenon disappears, and the dynamic responses of the microrobot show characteristics of single-period movement. This is mainly because a higher excitation frequency does not allow enough time for multi-contact interactions.

**Fig. 14:**
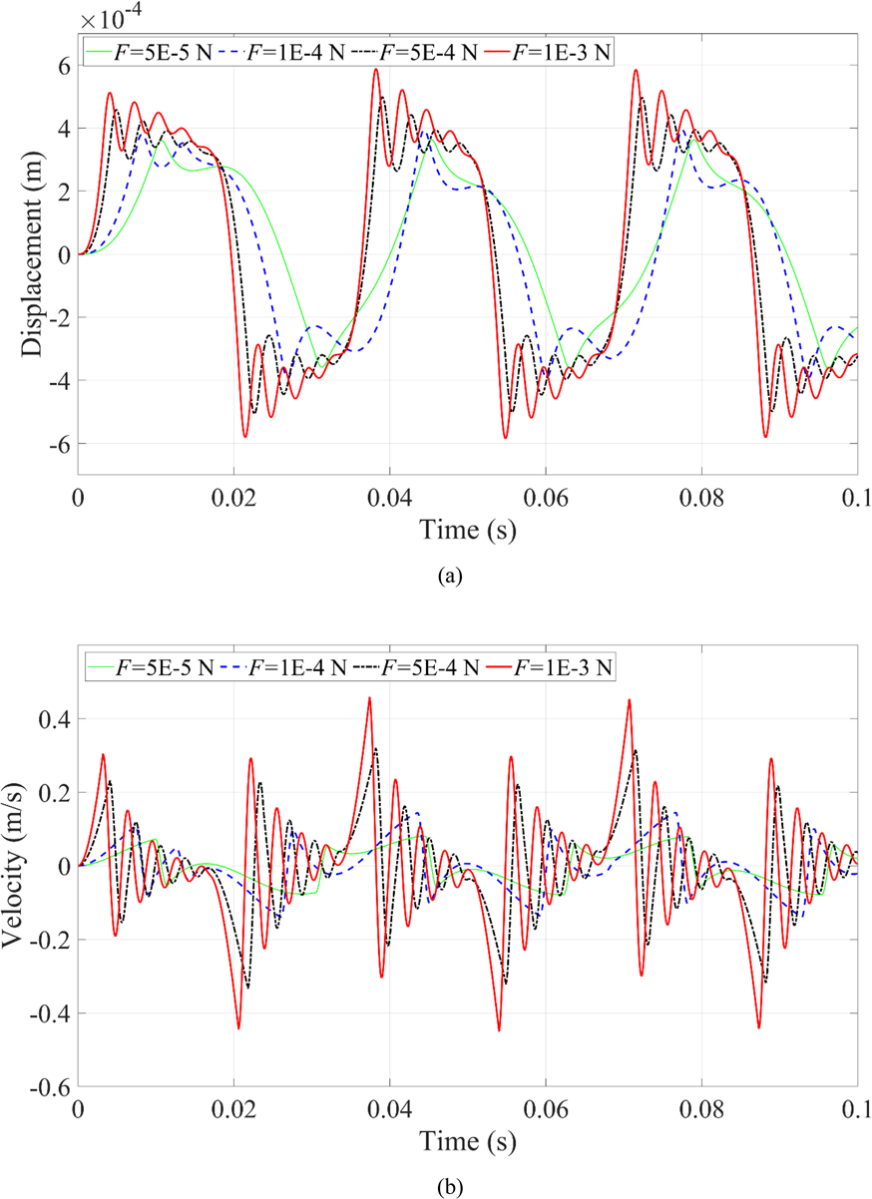
Dynamic responses of the microrobot under the variation of excitation force: **a** displacement of the microrobot; **b** velocity of the microrobot

Multiple collisions with different periodic motions are represented by multiple complete loops or circles with varying curvature in the phase portrait, as displayed in Fig. 13. The number of motion periods of the microrobot matches the number of the circles with different curvature in Fig. 13. The dashed black line represents the boundary of the blood vessel wall. When the curve exceeds this boundary, contact between the microrobot and the blood vessel wall occurs, and vice versa.

An interesting phenomenon is observed at 30 Hz, where a complete loop within the boundary appears in Fig. 13, representing a ‘fake’ collision behavior. This phenomenon is primarily influenced by the excitation frequency but is also related to the other relevant parameters. Due to the rich dynamic responses generated at 30 Hz, this frequency will be used to test the effects of other parameters on the dynamic responses of the microrobot in the following investigation.

**Fig. 15:**
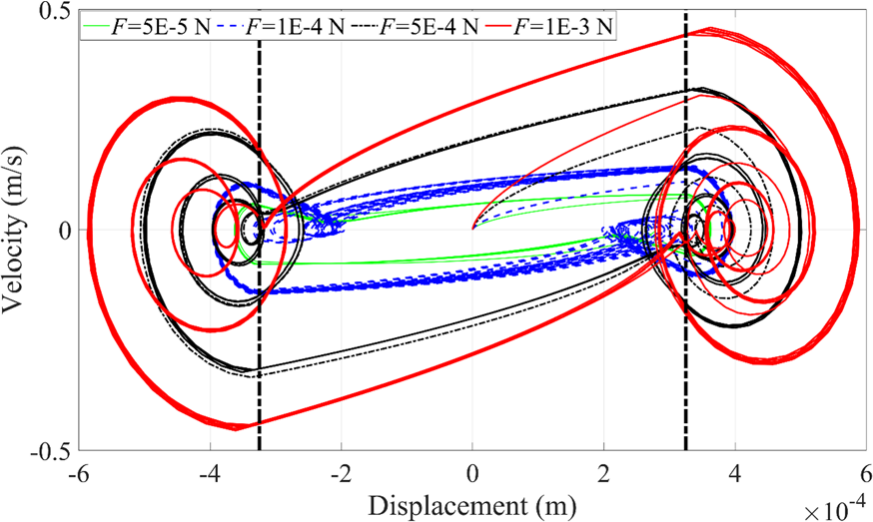
Phase trajectories of the microrobot under the variation of excitation force

The second scenario involves changing the magnitude of the excitation force; the dynamic responses of the microrobot can be seen in Figs. 14 and 15. When the excitation force increases, the multi-contact behavior between the microrobot and the blood vessel wall becomes more frequent and more obvious, as depicted in Figs. 14 and 15. Specifically, the characteristics of multi-periodic motion of the microrobot can be activated by increasing the excitation force, as shown in Fig. 15. In Fig. 14a, the displacement of the microrobot increases as the contact deformation between the microrobot and blood vessel wall enlarges. In Fig. 14b, the velocity of the microrobot increases because the acceleration of the microrobot rises with the excitation force. Obviously, the number of periodic motions included in the dynamic responses of the microrobot is closely related to the magnitude of the excitation force, increasing as the excitation force increases.

Likewise, by reducing the excitation force, the multi-periodic motion of the microrobot simplifies to a single periodic motion, as shown in Fig. 15. Nonetheless, in Fig. 15, it is clearly observed that the ‘fake’ contact behavior between the microrobot and the blood vessel wall disappears when the excitation force is too small (*F* = 5E-5 N) or too large (*F* = 1E-3 N). It is worth noting that the contact behaviors between the microrobot and the upper blood vessel wall differ from the contact behavior with the lower blood vessel wall. This is because the microrobot first contacts with the upper blood vessel wall under external harmonic excitation, and subsequently, the contact with the lower blood vessel wall will happen after more kinetic energy of the microrobot is dissipated by the fluid. Therefore, it is reasonable that the left side differs from the right side in the phase portrait as presented in Fig. 15, and this discrepancy between the left side and right sides in Fig. 15 will become more significant when the pertinent parameters reach certain values.

**Fig. 16:**
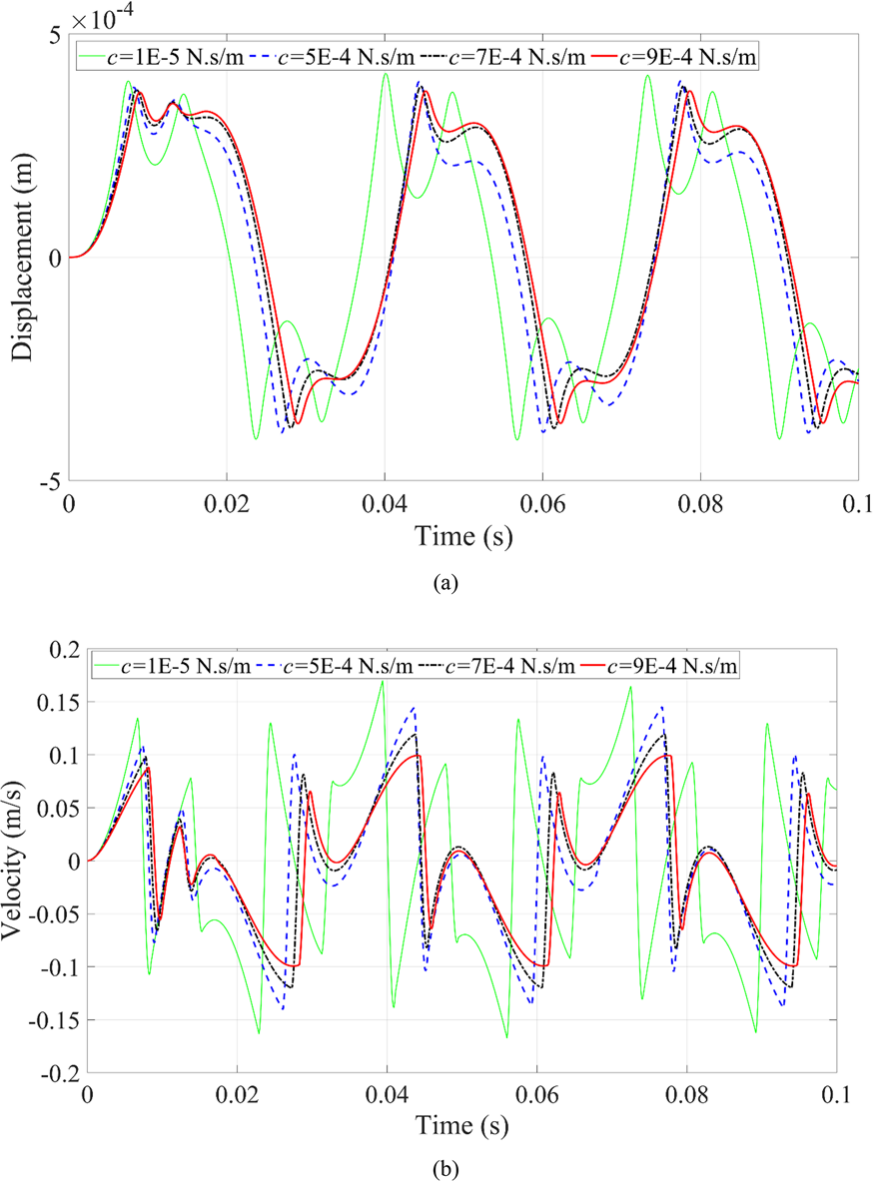
Dynamic responses of the microrobot under the variation of excitation frequency: **a** displacement of the microrobot; **b** velocity of the microrobot

**Fig. 17:**
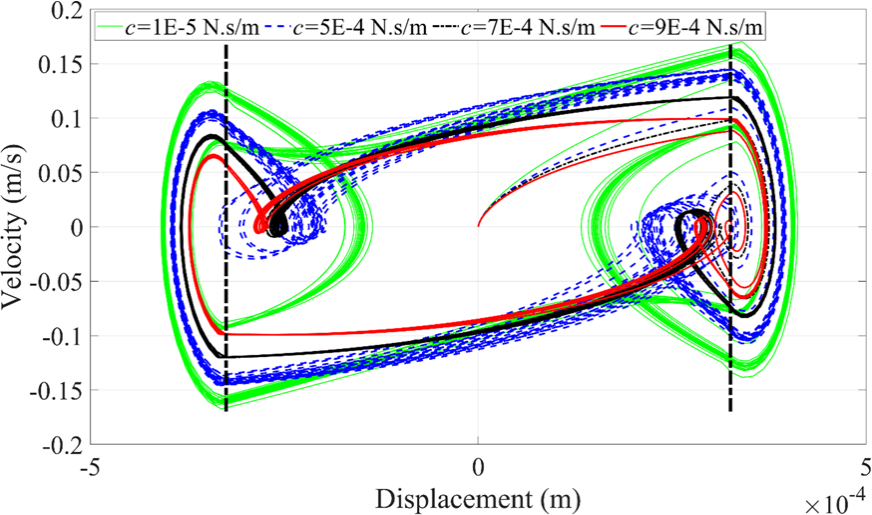
Phase trajectories under the variation of damping coefficient of fluid

The third scenario is to change the magnitude of the fluid damping coefficient. The dynamic responses of the microrobot can be seen in Figs. 16 and 17. This investigation employs a damping coefficient to simplify the effect of the complicated fluid on the dynamic responses of the microrobot moving in the fluid.

In Fig. 16, when the damping coefficient of the fluid has a greater value, the maximum displacement and velocity of the microrobot reduce simultaneously because the larger damping coefficient dissipates more energy, making the peak values of the dynamic responses of the microrobot decrease. Furthermore, a smaller fluid damping coefficient results in the dynamic responses of the microrobot having high efficiency feedback in comparison to the larger fluid damping coefficient as shown in Fig. 16.

The multi-periodic motion of the microrobot will degenerate into single periodic motion by increasing the fluid damping coefficient in Fig. 17. Moreover, the multi-periodic motion of microrobot near the upper blood vessel wall is more significant than that of the microrobot near the lower blood vessel wall in Fig. 17.

**Fig. 18:**
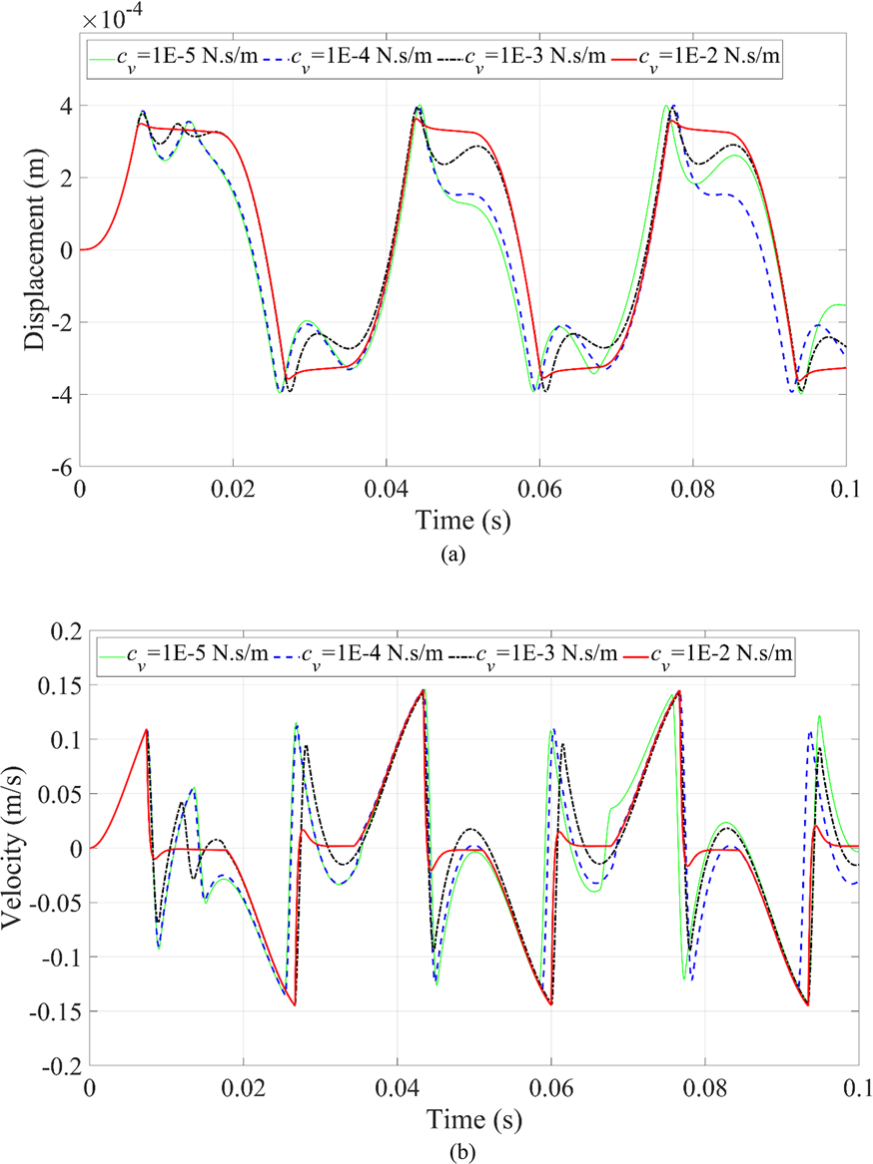
Dynamic responses of the microrobot under the variation of blood vessel damping coefficient: **a** displacement of the microrobot; **b** velocity of the microrobot

**Fig. 19:**
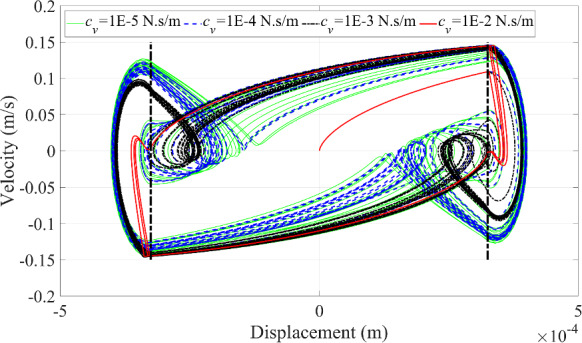
Phase trajectories under the variation of blood vessel damping coefficient

On the contrary, the ‘fake’ collision behavior between the microrobot and the blood vessel wall will disappear when reducing the damping coefficient (*c* = 1E-5 N·s/m) as demonstrated in Fig. 17.

The fourth scenario is to change the magnitude of the damping coefficient of the blood vessel wall. The dynamic responses of the microrobot can be seen in Figs. 18 and 19. When the damping coefficient of the blood vessel wall gradually increases until equal to 1E-2 N·s/m, the multi-periodic motion of the microrobot gradually degenerates into single period motion in Fig. 18a. A similar phenomenon can be clearly seen in the velocity of the microrobot in Fig. 18b. Therefore, the number of different periods in the dynamic responses of the microrobot can be identified by the number of curves of different curvatures crossing the boundary in Fig. 19.

**Fig. 20:**
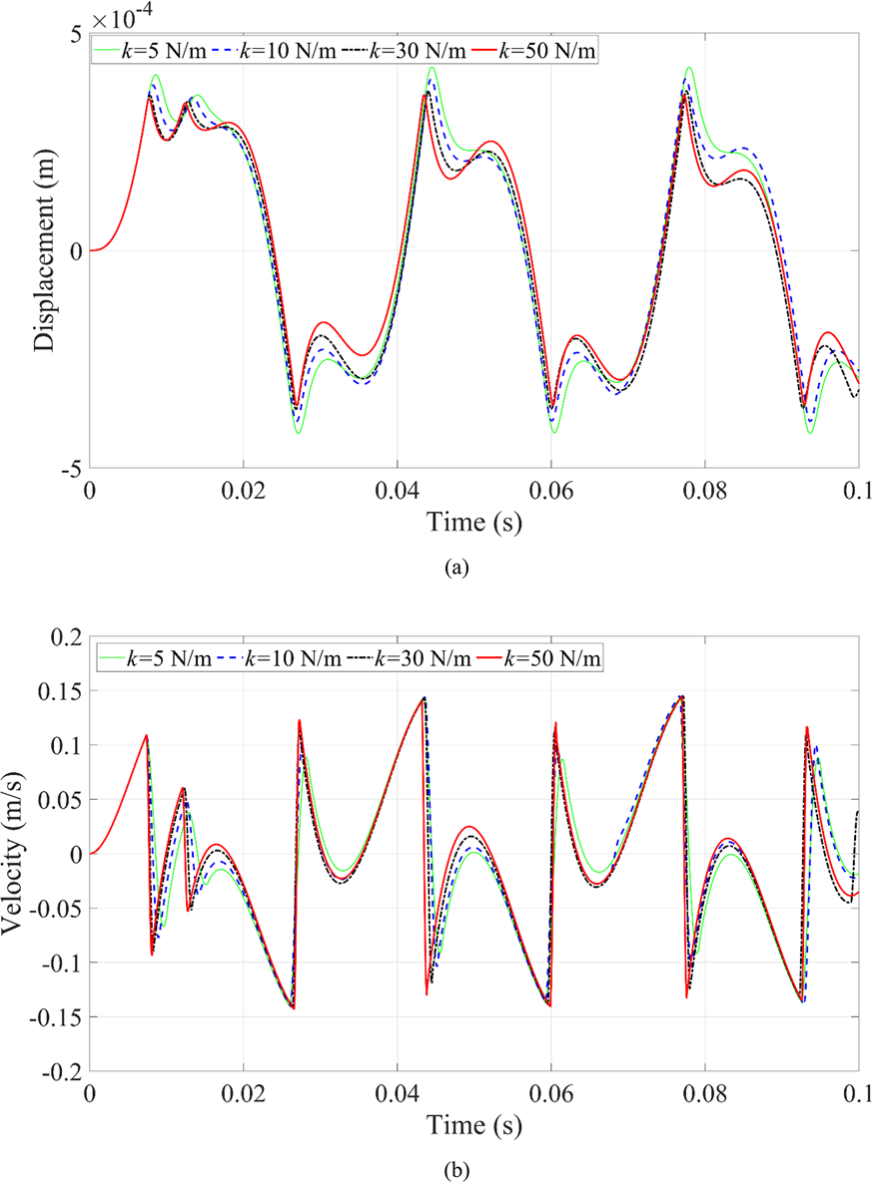
Dynamic responses of the microrobot under the variation of blood vessel stiffness coefficient: **a** displacement of the microrobot; **b** velocity of the microrobot

In addition to this, the ‘fake’ collision behavior disappears when the damping coefficient of the blood vessel wall is equal to 1E-2 N·s/m. Accordingly, the larger damping coefficient of the blood vessel wall can prevent the ‘fake’ collision behavior between the microrobot and the blood vessel wall.

**Fig. 21:**
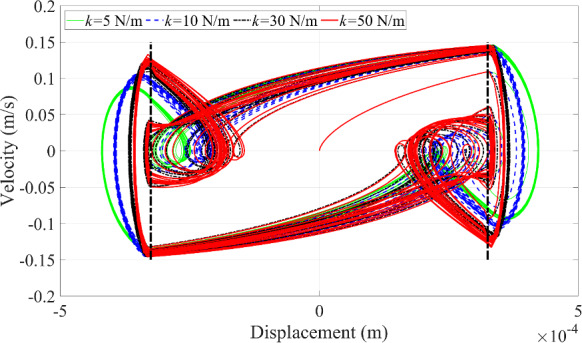
Phase trajectories under the variation of blood vessel stiffness coefficient

The fifth scenario is to change the magnitude of the stiffness coefficient of the blood vessel wall. The dynamic responses of the microrobot can be seen in Figs. 20 and 21. The features of the periodic motion of the microrobot are not sensitive to the stiffness of the blood vessel wall in Fig. 20 because the multi-period motion of the microrobot almost keeps consistent with each other when the stiffness of the blood vessel wall has different values. Only the magnitude of the displacement of the microrobot in Fig. 20a increases with the decreasing stiffness coefficient of the blood vessel wall because the smaller stiffness of the blood vessel wall is prone to deformation. The velocity of the microrobot in Fig. 20b is slightly different from each other when the stiffness coefficient of the blood vessel wall has different values.

Meanwhile, the different stiffness values of the blood vessel wall have no effect on the ‘fake’ collision behavior in Fig. 21. However, when the stiffness of the blood vessel wall increases, the radius of curvature of the circle also increases during contact between the microrobot and the blood vessel wall. This phenomenon occurs because the blood vessel wall becomes harder, thereby increasing the radius of curvature of the circle in Fig. 21, as the softer blood vessel wall is prone to deformation when contact behavior happens, as shown in Fig. 20a. The maximum displacement of the microrobot in Fig. 20a gradually increases with the decreasing stiffness of the blood vessel wall, leading to a gradual reduction in the radius of curvature of the circle in Fig. 21.

## Conclusions

This work formulated the simplified dynamics model of the microrobot vibrating in a blood vessel to detect potential cancer metastasis. To simplify the complexity of the fluid in blood vessel, a constant fluid damping coefficient was introduced to represent the fluid’s viscosity. In addition, a linear spring-dashpot model was used to describe the flexibility of the blood vessel wall and streamline the effect of its complicated deformation from microrobot impact on the dynamic responses of the entire system. According to these rational simplifications, linear one-degree-of-freedom vibration equations for the microrobot, with three different motion states under harmonic excitation, including contact behaviors between the microrobot and the upper blood vessel wall, free motion in fluid, and contact with the lower blood vessel wall, were formulated. An analytical solution of this system was solved based on the forced vibration theory and validated by experiments.

The transmitted force to the blood vessel wall is closely related to the damping and frequency ratios during contact behavior, dominated by the transmissibility function of these ratios. The greater the damping ratio, the smaller the force transmitted to the blood vessel wall. The transmitted force is larger than the original amplitude of the harmonic excitation when the frequency ratio is smaller than $\sqrt{2}$. Therefore, the magnitude of the transmitted force to the blood vessel wall is closely related to the internal damping and stiffness of the wall and determined by the external excitation frequency and amplitude. When contact behavior occurs and the frequency ratio equals 1, the resonance phenomenon of the microrobot is dominated by the magnification factor, which is a function of the damping ratio. The amplitude of the resonance behavior increases with decreasing damping ratio. Equation (29) shows that the resonance phenomenon is primarily controlled by the external excitation amplitude and internal damping coefficient of the blood vessel wall.

Eventually, the dynamic responses of the microrobot moving in the blood vessel are predicted under variations of related parameters, including excitation frequency and force, fluid damping, and stiffness and damping coefficients of the blood vessel wall. Two interesting phenomena, including multi-periodic motion and ‘fake’ collision behavior, were detected, independent of the stiffness coefficient of the blood vessel wall. Nonetheless, the multi-periodic motion of the microrobot is prone to activation when the excitation frequency, or fluid damping coefficient, or damping coefficient of the blood vessel wall has a relatively smaller value. On the contrary, the multi-periodic motion of the microrobot will degenerate into single period motion with reduced excitation force. Regarding the ‘fake’ collision behavior, it will disappear with increasing excitation frequency or damping coefficient of the blood vessel wall. Conversely, the ‘fake’ collision behavior will also disappear with decreasing fluid damping coefficient, and the behavior disappears with either too small or too large an excitation force.

The analytical solutions and simulated conclusions provided a solid theoretical foundation for developing the control strategy of the microrobot moving in the complex fluid environment. The ‘fake’ collision can be used to judge the magnitude of the excitation force because the small or larger excitation force makes the ‘fake’ collision disappear in Fig. 15. Moreover, the ‘fake’ collision behavior can also identify the viscosity of the fluid because it disappears when reducing the damping coefficient in Fig. 17. Conversely, when the damping coefficient of the blood vessel increase, the ‘fake’ collision will disappear in Fig. 19. In addition to this, the multi-contact phenomenon between the microrobot and vessel wall serves as the criteria to identify the scope of the excitation frequency in Fig. 12. As for the relationship between the displacement and velocity, the number of different periods in the dynamic responses of the microrobot can be identified by the number of the curves of different curvatures crossing the boundary. Further, when the stiffness of the blood vessel wall increases, the radius of curvature of the circle also increases during contact between the microrobot and the blood vessel wall in Fig. 21. These simulation results are helpful to allow for the determination of cancer metastasis. Future work will focus on bifurcation analysis using path-following methods to identify different microrobot motions, control strategy design, study of dynamics of multiple microrobots, and experimental validation.

## Data Availability

The numerical and experimental data sets generated and analyzed during the current study are available from the corresponding author upon reasonable request.

## Declarations

### Data accessibility

The numerical and experimental data sets generated and analyzed during the current study are available from the corresponding author upon reasonable request.

## References

[1] Tian, J., Afebu, K.O., Bickerdike, A., Liu, Y., Prasad, S., Nelson, B.J.: Fundamentals of bowel cancer for biomedical engineers. Ann. Biomed. Eng. **51**, 679–701 (2023). 10.1007/s10439-023-03155-8 36786901 10.1007/s10439-023-03155-8 PMC9927048

[2] Li, J., De Ávila, B.E., Gao, W., Zhang, L., Wang, J.: Micro / nanorobots for biomedicine: delivery, surgery, sensing, and detoxification. Sci. Robot **2**, 1–10 (2017) 10.1126/scirobotics.aam6431 PMC6759331 31552379

[3] Islam, Y., Leach, A.G., Smith, J., Pluchino, S., Coxon, C.R., Sivakumaran, M., Downing, J., Fatokun, A.A., Teixidò, M.: Physiological and pathological factors affecting drug delivery to the brain by Nanoparticles. Adv. Sci. **8**, 1–45 (2021). 10.1002/advs.202002085 10.1002/advs.202002085 PMC8188209 34105297

[4] Zhang, T., Liu, X., Qin, H., Lin, Y., Li, B., Jiang, X., Zheng, X.: Semiphysical design concept for developing miniaturized microrobots. Nano Lett. (2024). 10.1021/acs.nanolett.4c00025 10.1021/acs.nanolett.4c00025 38602330

[5] Chah, A., Belharet, K.: Tele-Guidance of a soft magnetic microrobot transported by a fluid in a vascular network. Actuators **12**, 1–19 (2023)

[6] Arcese, L., Cherry, A., Fruchard, M., Ferreira, A.: Optimal trajectory for a microrobot navigating in blood vessels. In: 32nd Annu, pp. 1950–1953. Int. Conf. IEEE EMBS Buenos Aires, Argentina (2010) 10.1109/IEMBS.2010.5627813 21097005

[7] Jeon, S., Hoshiar, A.K., Kim, K., Lee, S., Kim, E., Lee, S., Kim, J., Nelson, B.J., Cha, H., Yi, B.-J., Choi, H.: A magnetically controlled soft microrobot steering a guidewire in a three-dimensional. Soft Robot **6**, 54–68 (2019). 10.1089/soro.2018.0019 30312145 10.1089/soro.2018.0019 PMC6386781

[8] Sa, J., Park, J., Jung, E., Kim, N., Lee, D., Bae, S., Lee, Y., Jang, G.: Separable and recombinable magnetic robot for robotic endovascular intervention, IEEE robot. Autom. Lett. **8**, 1881–1888 (2023). 10.1109/LRA.2023.3243801

[9] Damak, M., de Ruiter, J., Panat, S., Varanasi, K.K.: Dynamics of an impacting emulsion droplet. Sci. Adv. **8**, 1–10 (2022). 10.1126/sciadv.abl7160 10.1126/sciadv.abl7160 PMC8932654 35302841

[10] Lavergne, F.A., Wendehenne, H., Bäuerle, T., Bechinger, C.: Group formation and cohesion of active particles with visual perception–dependent motility Science **364**, 70–74 (2019). 10.1126/science.aau5347 30948548 10.1126/science.aau5347

[11] Hochella, M.F., Mogk, D.W., Ranville, J., Allen, I.C., Luther, G.W., Marr, L.C., McGrail, B.P., Murayama, M., Qafoku, N.P., Rosso, K.M., Sahai, N., Schroeder, P.A., Vikesland, P., Westerhoff, P., Yang, Y.: Natural, incidental, and engineered nanomaterials and their impacts on the earth system. Science (2019). 10.1126/science.aau8299 10.1126/science.aau8299 30923195

[12] Zhang, R., Luo, W., Zhang, Y., Zhu, D., Midgley, A.C., Song, H., Khalique, A., Zhang, H., Zhuang, J., Kong, D., Huang, X.: Particle-based artificial three-dimensional stem cell spheroids for revascularization of ischemic diseases. Sci. Adv. **6**, 1–14 (2020). 10.1126/sciadv.aaz8011 10.1126/sciadv.aaz8011 PMC7202876 32494716

[13] Nandihalli, N., Gregory, D.H., Mori, T., Solvothermal, M.-A.: Solution-based, and powder processing. Adv. Sci. **9**, 1–61 (2022). 10.1002/advs.202106052 10.1002/advs.202106052 PMC9443476 35843868

[14] Sharif, S., Jung, D., Cao, H.X., Park, J., Kang, B., Choi, E.: Ultrasonic manipulation of hydrodynamically driven microparticles in vessel bifurcation: simulation, optimization, experimental validation, and potential for targeted drug delivery. Micromachines **15**, 1–14 (2024) 10.3390/mi15010013 PMC10819303 38276841

[15] Bizmark, N., Schneider, J., Priestley, R.D., Datta, S.S.: Multiscale dynamics of colloidal deposition and erosion in porous media. Sci. Adv. **6**, 1–11 (2020). 10.1126/sciadv.abc2530 10.1126/sciadv.abc2530 PMC7673751 33188022

[16] Stogin, B.B., Gockowski, L., Feldstein, H., Claure, H., Wang, J., Wong, T.S.: Free-standing liquid membranes as unusual particle separators. Sci Adv. (2018). 10.1126/sciadv.aat3276 10.1126/sciadv.aat3276 PMC6108570 30151426

[17] Li, T., Yu, S., Sun, B., Li, Y., Wang, X., Pan, Y., Song, C., Ren, Y., Zhang, Z., Grattan, K.T.V., Wu, Z., Zhao, J.: Bioinspired claw-engaged and biolubricated swimming microrobots creating active retention in blood vessels. Sci. Adv. **9**, 1–16 (2023). 10.1126/sciadv.adg4501 10.1126/sciadv.adg4501 PMC10162671 37146139

[18] Bozuyuk, U., Ozturk, H., Sitti, M.: Microrobotic locomotion in blood vessels: a computational study on the performance of Surface microrollers in the cardiovascular system. Adv. Intell. Syst. **5**, 1–12 (2023). 10.1002/aisy.202300099

[19] Jeong, S., Choi, H., Go, G., Lee, C., Seob, K., Sun, D., Ho, M., Young, S., Park, J., Park, S.: Penetration of an artificial arterial thromboembolism in a live animal using an intravascular therapeutic microrobot system. Med. Eng. Phys. **38**, 403–410 (2016). 10.1016/j.medengphy.2016.01.001 26857290 10.1016/j.medengphy.2016.01.001

[20] Sun, Y., Fruchard, M., Ferreira, A.: Adaptive replanning and control for magnetic microrobots tracking despite unknown blood velocity, Proc. IEEE Conf. Decis. Control 2021-Decem 1789–1794. (2021). 10.1109/CDC45484.2021.9683665

[21] Talaśka, K., Ferreira, A.: An Approach to identifying phenomena accompanying micro and nanoparticles in contact with irregular vessel walls. IEEE Trans. Nanobiosci. **16**, 463–475 (2017). 10.1109/TNB.2017.2717178 10.1109/TNB.2017.2717178 28641266

[22] Liu, Y., Páez, J., Chávez: Controlling coexisting attractors of an impacting system via linear augmentation. Phys. D Nonlinear Phenom. **348**, 1–11 (2017). 10.1016/j.physd.2017.02.018

[23] Zhang, Z., Páez Chávez, J., Sieber, J., Liu, Y.: Controlling grazing-induced multistability in a piecewise-smooth impacting system via the time-delayed feedback control. Nonlinear Dyn. **107**, 1595–1610 (2022). 10.1007/s11071-021-06511-2

[24] Ing, J., Pavlovskaia, E., Wiercigroch, M., Banerjee, S.: Experimental study of impact oscillator with one-sided elastic constraint experimental study of impact oscillator with one-sided elastic constraint. Philos. Trans. R Soc. Math. Phys. Eng. Sci. **366**, 679–705 (2008). 10.1098/rsta.2007.2122 10.1098/rsta.2007.2122 17947209

[25] Zhang, Z., Páez, J., Sieber, J., Liu, Y.: Numerical analysis of a multistable capsule system under the delayed feedback control with a constant delay. Int. J. Non Linear Mech. **152**, 104390 (2023). 10.1016/j.ijnonlinmec.2023.104390

[26] Yin, S., Yan, Y., Páez Chávez, J., Liu, Y.: Dynamics of a self-propelled capsule robot in contact with different folds in the small intestine. Commun. Nonlinear Sci. Numer. Simul. **126**, 107445 (2023). 10.1016/j.cnsns.2023.107445

[27] Guo, B., Páez, J., Liu, Y., Liu, C.: Discontinuity-induced bifurcations in a piecewise-smooth capsule system with bidirectional drifts. Commun. Nonlinear Sci. Numer. Simul. **102**, 105909 (2021). 10.1016/j.cnsns.2021.105909

[28] Wang, J., Schwenger, J., Ströbel, A., Feldner, P., Herre, P., Romeis, S., Peukert, W., Merle, B., Vogel, N.: Mechanics of colloidal supraparticles under compression. Sci. Adv. (2021). 10.1126/sciadv.abj0954 10.1126/sciadv.abj0954 PMC11095630 34644116

[29] Fringand, T., Cheylan, I., Lenoir, M., Mace, L., Favier, J.: A stable and explicit fluid–structure interaction solver based on lattice-boltzmann and immersed boundary methods. Comput. Methods Appl. Mech. Eng. **421**, 116777 (2024). 10.1016/j.cma.2024.116777

[30] Wang, S., Ardekani, A.M.: Unsteady swimming of small organisms. J. Fluid Mech. **702**, 286–297 (2012). 10.1017/jfm.2012.177

[31] Tabak, A.F., Yesilyurt, S.: Computationally-validated surrogate models for optimal geometric design of bio-inspired swimming robots: HELICAL swimmers. Comput. Fluids. **99**, 190–198 (2014). 10.1016/j.compfluid.2014.04.033

[32] Hosney, A., Abdalla, J., Amin, I.S., Hamdi, N., Khalil, I.S.M.: In vitro validation of clearing clogged vessels using microrobots, In: 6th IEEE RAS/EMBS int. Conf. Biomed. Robot Biomechatronics, : pp. 1–6. (2016)

[33] Barbeau, L., Golshan, S., Deng, J., Étienne, S., Béguin, C., Blais, B.: High-order moving immersed boundary and its application to a resolved CFD-DEM model. Comput. Fluids. **268**, 106094 (2024). 10.1016/j.compfluid.2023.106094

[34] Li, Q., Abbas, M., Morris, J.F.: Particle approach to a stagnation point at a wall: viscous damping and collision dynamics. Phys. Rev. Fluids **5**, 104301 (2020). 10.1103/PhysRevFluids.5.104301

[35] Nagata, T., Hosaka, M., Takahashi, S., Shimizu, K., Fukuda, K., Obayashi, S.: A simple collision algorithm for arbitrarily shaped objects in particle-resolved flow simulation using an immersed boundary method. Int. J. Numer. Methods Fluids. **92**, 1256–1273 (2020). 10.1002/fld.4826

[36] Erman, A.G., Tabak, A.F.: Resistive force theory based modeling and simulation of surface contact for swimming helical micro robots with channel flow, IEEE/ASME int. Conf. Adv. Intell. Mechatronics AIM. 390–395 (2014). 10.1109/AIM.2014.6878110

[37] Tabak, A.F., Temel, F.Z., Yesilyurt, S.: Comparison on experimental and numerical results for helical swimmers inside channels. IEEE Int. Conf. Intell. Robot Syst. (2011). 10.1109/IROS.2011.6048187

[38] Peng, Q., Wang, S., Han, J., Huang, C., Yu, H., Li, D., Qiu, M., Cheng, S., Wu, C., Cai, M., Fu, S., Chen, B., Wu, X., Du, S., Xu, T.: Thermal and magnetic dual-responsive catheter-assisted shape memory microrobots for multistage vascular embolization. Research. **7**, 1–15 (2024). 10.34133/research.0339 10.34133/research.0339 PMC10976590 38550780

[39] Zhong, Z., Niu, F., Li, J., Wang, J., Huan, Z., Ma, W., Wu, F.: Path planning and optimization for micro-robot in a vessel-mimic environment. Front. Neurorobot **16**, 1–7 (2022) 10.3389/fnbot.2022.923348 PMC9489927 36160285

